# Phytochemical Dynamics and Antimicrobial Efficacy of Dandelion (*Taraxacum officinale* L.) from Central Plateau of Moldova, Romania

**DOI:** 10.3390/molecules31142549

**Published:** 2026-07-22

**Authors:** Maria-Virginia Tanasa (Acretei), Ticuta Negreanu-Pirjol, Verginica Schröder, Laura Adriana Bucur, Bogdan-Stefan Negreanu-Pirjol, Florentina Nicoleta Roncea, Antoanela Popescu, Ioana Cristina Marinas, Diana-Madalina Gaboreanu, Dan Razvan Popoviciu, Simona Margareta Coman, Natalia Rosoiu

**Affiliations:** 1 Doctoral School of Applied Sciences, ISD Institute of Doctoral Studies, Ovidius University of Constanta, 900573 Constanta, Romania; maria.acretei@365.univ-ovidius.ro (M.-V.T.); natalia_rosoiu@yahoo.com (N.R.); 2 Pharmaceutical Sciences Department, Faculty of Pharmacy, Ovidius University of Constanta, 900470 Constanta, Romania; laurabucur@univ-ovidius.ro (L.A.B.); bogdan.negreanu@univ-ovidius.ro (B.-S.N.-P.); florentina.roncea@univ-ovidius.ro (F.N.R.); antoanela.popescu@univ-ovidius.ro (A.P.); 3 Biological Sciences Section, Academy of Romanian Scientists, 050045 Bucharest, Romania; 4 Doctoral School of Pharmacy, Institute of Doctoral Studies, Ovidius University of Constanta, 900573 Constanta, Romania; 5 Research Institute of the University of Bucharest, 050095 Bucharest, Romania; ioana.cristina.marinas@gmail.com (I.C.M.); gaboreanu.diana-madalina@s.bio.unibuc.ro (D.-M.G.); 6 Botany and Microbiology Department, Faculty of Biology, University of Bucharest, 050095 Bucharest, Romania; 7 Faculty of Natural Sciences and Agricultural Sciences, Ovidius University of Constanta, 900470 Constanta, Romania; dr_popoviciu@yahoo.com; 8 Department of Inorganic & Organic Chemistry, Biochemistry and Catalysis, Faculty of Chemistry, University of Bucharest, 030018 Bucharest, Romania; simona.coman@chimie.unibuc.ro; 9 Faculty of Medicine, Ovidius University of Constanta, 900470 Constanta, Romania

**Keywords:** *Taraxacum officinale* L., dandelion, hydroalcoholic extracts, bioactive compounds, seasonal variation, phytochemical profiling, antimicrobial activity, matrix effect

## Abstract

The therapeutic use of *Taraxacum officinale* L. remains a challenge due to its chemical profile shifting dramatically, markedly with seasonal variation and processing techniques. To address this, the paper evaluated how the plant organ, harvest season, and extraction technique as ultrasound-assisted extraction (UAE) and solid–liquid extraction in a Soxhlet system (both using hydroalcoholic solvent concentrations of 70:30 (*v*/*v*) ethanol) and conventional cold maceration, at solvent concentration 50:50 (*v*/*v*) ethanol, respectively 70:30 (*v*/*v*) ethanol, could have impact both metabolite yield and biological activity. The findings show that a single, uniform extraction protocol is inefficient; instead, the data support a dual-harvest approach of vegetal product. Autumn harvests are ideal for extracting tannins and anthocyanins, while spring harvests maximize flavonoids, ascorbic acid, and carotenoids. In terms of methodology, UAE, with 70:30 (*v*/*v*) ethanol, consistently outperforms other approaches because it prevents thermal degradation of Soxhlet extraction and improves the recovery of intermediate-polarity compounds. As a result, UAE extracts showed the strongest antimicrobial action, particularly against Gram-positive bacteria such as *Staphylococcus aureus*, and notable effectiveness against *Pseudomonas aeruginosa*. In addition, brine shrimp lethality screening confirmed the safety of all extracts (LC_50_ > 1000 µg/mL). Notably, the root extracts induced a specific, non-lethal delay in larval development, likely tied to their unique bitter principles. This research provides a practical framework for tailoring harvest and extraction parameters to target specific compounds for clinical and nutraceutical use.

## 1. Introduction

**Figure 1:**
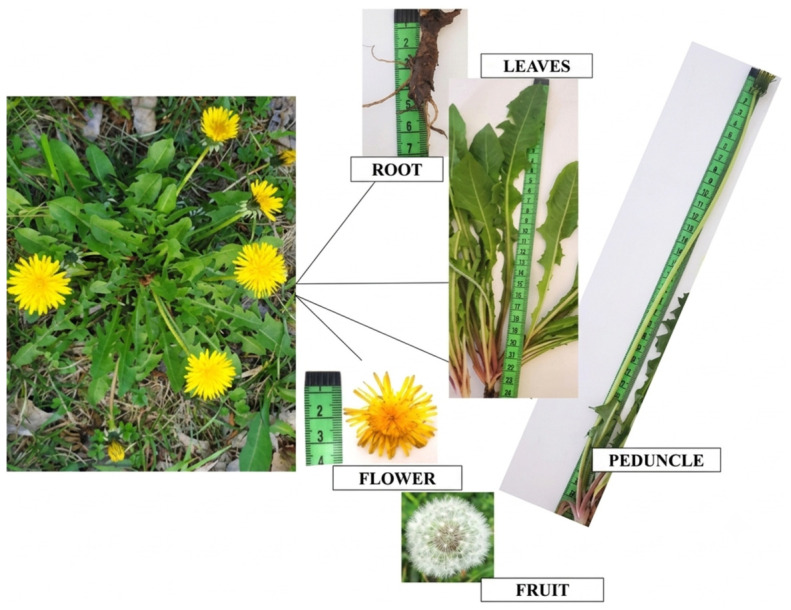
*Taraxacum officinale* L. vegetal organs (M.-V.T., original photos).

*Taraxacum* species, commonly known as dandelions, are versatile members of the *Asteraceae* family. Native to Eurasia, but now widely distributed across warm-temperate zones—ranging from sea level to alpine elevations—this genus is remarkably diverse, with the International Plant Names Index (2026) recognizing over 3672 species. The plant is easily identified by its distinct physical structure [1]. It possesses a deep, sturdy taproot and a very short stem supporting a basal rosette of leaves (Figure 1), which can vary from smooth-margined to deeply serrated, and produces solitary yellow flower heads composed of ray florets. These mature into characteristic ball-shaped clusters of tufted, one-seeded fruits designed for wind dispersal. Naturally growing *Taraxacum* species reflect their local climatic conditions and are known for their resilience, supported by a robust enzymatic and non-enzymatic system developed as a mechanism for stress tolerance [2].

### 1.1. Phytochemical Analysis

Modern research often uses high-sensitivity platforms such as High-Performance Liquid Chromatography (HPLC) and Nuclear Magnetic Resonance (NMR) spectroscopy, alongside mass spectrometry-based hybrids like LC-ESI-MS and UHPLC/HR-QTOF-MS for the detailed profiling of complex plant matrices [3]. Practical applications of these technologies include the successful isolation of key phenolic compounds—such as chlorogenic acid, caffeic acid, luteolin, and luteolin-7-O-glucoside—across various botanical fractions [4]. Advanced isolation techniques have also led to the detection of unique molecules within the genus, including previously undocumented guaianolide glucosides isolated from *Taraxacum obovatum* [5].

Recent studies highlight that the chemical profile of *T. officinale* L. is subject to significant fluctuations dictated by both geographic provenance and solvent extraction parameters. For instance, comparisons between specimens from distinct bioregions demonstrate substantial regional disparities in flavonoid accumulation, driven by the local ecology, soil geochemistry, and microclimate [6]. Furthermore, the quantitative distribution of bioactive compounds exhibits significant organ-specific variation. Studies on *T. officinale* populations show distinct partitioning of carotenoids and ascorbic acid between foliar tissues and floral organs [7], while genus-wide perspectives on *Taraxacum mongolicum* indicate that the hierarchy of phenolic accumulation often prioritizes reproductive structures [8]. The broader impact of environmental adaptation and cultivation methods on the phenolic profile confirms that distinct ecotypes exhibit superior accumulation of primary polyphenols, such as chicoric, caftaric, chlorogenic, and caffeic acids [9]. This aligns with established botanical literature positing a generalized trend wherein the aerial organs of *Taraxacum* species possess a more robust polyphenolic density compared to their subterranean counterparts [10,11]. Additionally, the extraction yield of these metabolites remains highly contingent upon the polarity of the solvents employed, with varying recovery efficiencies observed between non-polar media, polar solvents, and aqueous extracts [12].

### 1.2. Biometals, Trace Elements and Heavy Metals Content in Taraxacum officinale L. Relative to Plant Organ and Pedo-Climatic Conditions

*Taraxacum officinale* L. is a ubiquitous species characterized by a high capacity for the uptake and bioaccumulation of mineral elements. This renders it a significant subject for both nutritional assessment and ecotoxicological monitoring. Its mineral profile is governed by multifactorial drivers, including organ-specific allocation, pedological soil characteristics, and local anthropogenic pressure. Current literature indicates that leaves serve as the primary reservoir for macro elements, whereas roots more accurately reflect the soil’s chemical composition regarding heavy metal concentrations (European Medicines Agency, 2021) [13].

Comparative studies of mineral distribution reveal a highly differentiated partitioning of biometals across leaves, stems, flowers, and roots [14]. On a global scale, these baselines vary drastically depending on local conditions, spanning from standard median ranges in urban settings [15], to highly elevated accumulations near active mining and industrial sites [16].

Recent research reinforces the status of *T. officinale* L. as an exceptional bioindicator for passive environmental monitoring. Studies in urban centers (Warsaw, Poznań, Wrocław) demonstrate that heavy metal accumulation in leaves varies significantly (Table 1) with land use, with traffic emissions playing a decisive role [17]. Furthermore, massive accumulations of iron and manganese are frequently recorded near industrial steelworks [18], while standard-exceeding concentrations of cadmium near mining areas illustrate highly efficient soil-to-plant pollutant [19].

The relationship between plant organs and element mobility is critical; while leaves reflect both root uptake and atmospheric deposition, roots remain a more faithful indicator of soil-specific contamination.

**Figure 2:**
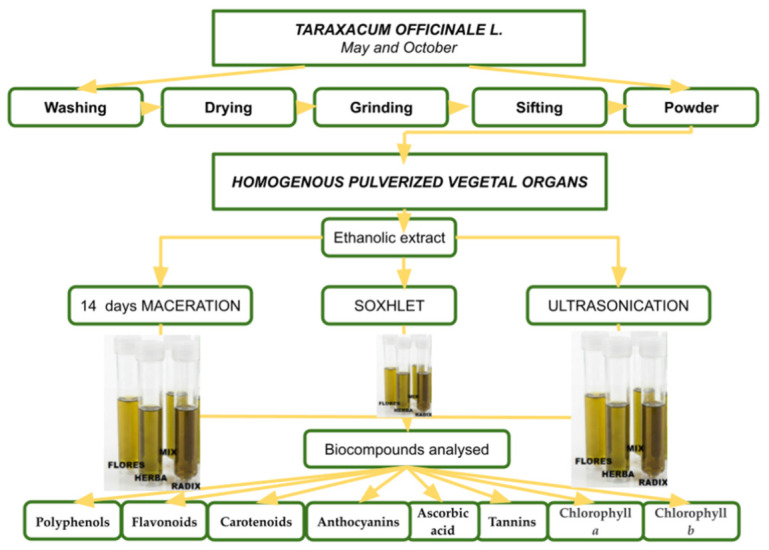
Process design to identify and obtain bioactive compounds from *Taraxacum officinale* L.

*T. officinale* L. specimens were collected in May and October. The harvested organs were washed, dried, ground, and sifted to yield homogeneous powders for each anatomical part, and named: Radix, Herba, Flores, Mix. To compare extraction efficiencies, the matrices underwent parallel ethanolic extractions for 14 days at ambient temperature maceration using 50:50 (*v*/*v*) and 70:30 (*v*/*v*) ethanol, Soxhlet extraction, and ultrasound-assisted extraction (UAE) (Soxhlet and UAE used 70:30 (*v*/*v*) ethanol). The resulting extracts were characterized through a multi-tiered analytical approach: initial qualitative phytochemical screening was followed by quantitative physicochemical profiling of specific biocompounds (Figure 2) using UV-vis spectrophotometry and HPLC-DAD. Concurrently, essential biometals and heavy metals were quantified to evaluate seasonal bioaccumulation dynamics. Finally, the biological potential of the extracts was validated via in vitro antimicrobial evaluations and cytotoxicity testing using the *Artemia salina* lethality bioassay. 

## 2. Results

### 2.1. Phytochemical Analysis of Vegetal Organs

#### 2.1.1. Evaluation of Herbal Extracts: Radix, Herba, Flores and Mix

The physicochemical characterization of herbal extracts is a fundamental step in the standardization of phytotherapeutic products. To accurately evaluate the efficiency of the extraction methods (maceration, ultrasound-assisted extraction, and Soxhlet) and the quality of the resulting *T. officinale* L. phytocomplex, an analysis of four essential macroscopic parameters of hydroalcoholic extracts was conducted, including pH, electrical conductivity, refractive index (%Brix), and density. These indicators were selected because, together, they provide a comprehensive identification of the extraction process’s thermodynamic and compositional dynamics:

- pH and density are critical parameters for predicting the long-term stability of the fluid extracts, preventing the early oxidation of phenolic compounds, and determining their safety and compatibility for subsequent topical formulations.
- electrical conductivity quantifies the ionic and mineral load of the solution, acting as a reliable specific marker for the electrolytic contribution of the plant’s aerial parts.
- refractive index (%Brix) serves as a direct indicator of the extraction yield, reflecting the concentration of soluble dry matter (such as inulin, sugars, and secondary metabolites) transferred into the solvent.

**Figure 3:**
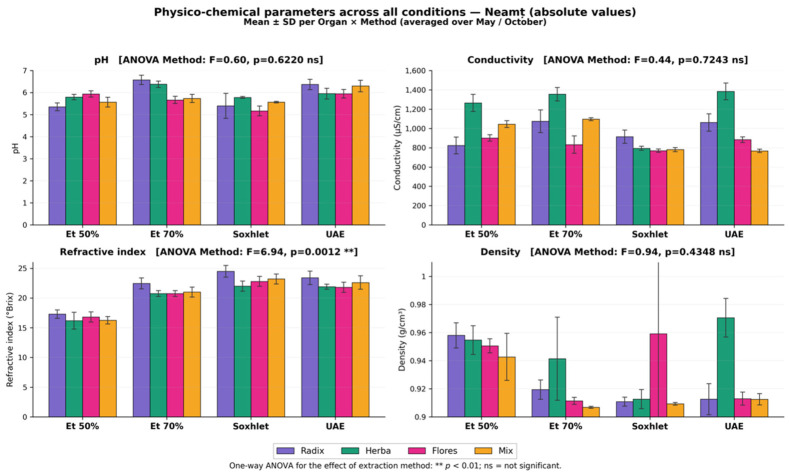
Physicochemical parameters of *T. officinale* L. hydroalcoholic extracts according to plant part and extraction methodology. Note: Et 50% = ambient temperature maceration in ethanol 50:50 (*v*/*v*), Et 70% = ambient temperature maceration in ethanol 70:30 (*v*/*v*), UAE = ultrasound-assisted extraction, ns = not significant, ** *p* < 0.001.

The analysis of extract stability and extraction methods presents and critically discusses variations in these parameters with respect to the specific plant organ and harvesting season, thereby establishing the technological foundation for selecting the optimal extraction matrix (Figure 3).

The recorded pH values (ranging from 5.00 to 6.72) indicate a moderately acidic profile, typical for extracts rich in phenolic acids. A slight decrease in pH was observed in several October samples (e.g., Radix Oct Sox at 5.00). This acidification is probably related to the accumulation of polyphenols and tannins in the autumn root. These compounds, particularly chlorogenic and caffeic acid derivatives, contribute to the antioxidant capacity and chemical acidity of the late-season extracts. Conductivity (μS/cm) measurements provided a quantitative proxy for the ionization of mineral content within the hydroalcoholic medium.

The results revealed a significant spring peak in the Radix and Herba categories. For instance, Radix May 70% reached 1156 μS/cm, surpassing the Radix Oct 70% value of 991 μS/cm. This physical parameter aligns with our elemental analysis, which showed a massive mobilization of Iron (Fe) and Manganese (Mn) during May. This reflects the plant’s metabolic shift: in spring, minerals are actively ionized and translocated to the aerial parts to facilitate chlorophyll biosynthesis and enzymatic catalysis. The higher conductivity in May extracts validates the superior remineralizing potential of spring-harvested dandelion.

Determination of the refractive index served as a primary indicator for the total concentration of dissolved solids, including carbohydrates and secondary metabolites. A distinct seasonal trend was observed in the root samples (Radix), where October extracts consistently exhibited higher values compared to May (e.g., Radix Oct Sox at 25.2% vs. Radix May Sox at 23.8%). The data indicate that UAE and Soxhlet extraction (Sox) yielded significantly higher soluble solids compared to traditional maceration (50% and 70% ethanol). Soxhlet extraction provided the highest density in inflorescences (Flores May Sox: 1.01071 (g/cm^3^), suggesting that the intensive thermal cycle successfully extracted complex lipophilic pigments and waxes. UAE maintained a high conductivity and refractive index while operating at lower temperatures, potentially preserving thermolabile compounds such as ascorbic acid, which our previous analysis identified as being at maximum concentration in spring.

To evaluate the influence of the sampling period and extraction method on the physicochemical properties of the hydroalcoholic extracts, a statistical analysis of variance was conducted. The results indicate that both period and method are critical determinants for most, but not all, of the evaluated parameters. Specifically, the sampling period exerted a highly significant (*p* < 0.001) main effect on the extracts, density, and refractive index (Table 2).

The extraction method also strongly influenced these same physical characteristics, with a particularly marked effect (*p* < 0.001) on the refractive index, and significant effects on density and pH. In contrast, conductivity of the samples remained stable regardless of the variables tested; neither the sampling period (*p* < 0.181) nor the extraction method (*p* < 0.130) produced any statistically significant changes in this specific parameter.

#### 2.1.2. Comparative Analysis of Mineral and Heavy Metals Content of Vegetal Extracts

The analysis of the metal content revealed an uneven distribution of microelements depending on the analyzed plant organ (roots, aerial parts, flowers) and the harvest period (October vs. May). The values obtained reflect the concentration of elements in the analyzed solution, expressed in mg/L. Iron (Fe 248) presents the highest concentration of all samples. A significant increase in iron concentration is observed in the spring period (May) for individual organs: in the root, the level increases from 3.896 mg/L (October) (Oct) to 4.473 mg/L (May), and in the aerial parts (Herba) from 1.769 mg/L to 3.805 mg/L (Table 3).

This massive mobilization of iron in spring is correlated with the resumption of the vegetative cycle and the intense synthesis of assimilatory pigments (chlorophyll), for which iron is an essential cofactor.

A particularly interesting observation is the accumulation of zinc in the root (Radix) during autumn (October), registering 2.503 mg/L, much higher than any other sample. In spring (May), the roots value drops dramatically to 0.3207 mg/L. This phenomenon indicates the root’s role as a storage organ during winter, with microelements subsequently translocated to the aerial parts in spring.

Both elements, Mn 279 and Cu 324, present higher values in May compared to October for most individual organs (Radix, Herba, Flores). For example, the concentration of copper in inflorescences increases from 0.0784 mg/L (October) to 0.1108 mg/L (May). These elements are actively involved in oxidation-reduction processes and in the plant’s antioxidant protection during the flowering period and at maximum solar exposure.

From the perspective of plant product quality control, the results obtained for toxic metals are favorable and support the safety of using *T. officinale* L. samples. Lead (Pb 283) and Cadmium (Cd 228): all values recorded for these contaminants are negative (e.g., −0.1939 mg/L for Pb in Radix May; −0.0070 mg/L for Cd in Herba May). In instrumental analysis, negative values indicate concentrations below the device’s limit of detection (LOD). This demonstrates the absence of heavy-metal contamination of the soil from which the plants were harvested. Nickel (Ni 232): similar to lead and cadmium, nickel presents negative values in most organs, indicating its absence from the analyzed tissues. Only extremely fine traces are observed in inflorescences (0.0030 mg/L in October and 0.0073 mg/L in May), values that are negligible from a toxicological point of view.

The data confirms that the harvesting period dictates the mineral profile of the *T. officinale* L. species. Autumn harvesting favors the extraction of roots rich in zinc, while spring harvesting provides aerial parts and roots with a higher intake of iron and manganese. In addition, the absence of detectable toxic metals confirms the purity and suitability of the plant material for possible pharmaceutical preparations or extracts.

The integration of physicochemical parameters and mineral profiles confirms that the harvesting season determines the therapeutic profile of the extract. Spring harvesting provides a mineral- and vitamin-rich product (high Fe, Mn, and Vitamin C) with superior conductivity, suitable for remineralization. Conversely, autumn harvesting yields a nutritionally dense extract characterized by high %Brix and complex polyphenols, primarily driven by the accumulation of inulin and secondary metabolites in the root system. This is attributable to the ontogenetic cycle of *T. officinale* L. During late autumn, the plant translocates photosynthates from the aerial parts to the taproot, primarily in the form of inulin, a fructan-type polysaccharide.

#### 2.1.3. General Phytochemical Screening of Roots, Leaves and Inflorescences Extracts

The qualitative chemical assessment of *T. officinale* L. was performed via successive selective extraction. The pulverized vegetal material was subjected to a polarity-guided fractionation process utilizing three solvent systems: ethyl ether (apolar), ethanol (medium polarity), and distilled water (polar) (Table 4). This procedure resulted in the isolation of three distinct fractions: an ethereal fraction containing lipophilic constituents, and ethanolic and aqueous fractions concentrating hydrophilic principles. Subsequent identification of active compounds was achieved through specific chromogenic and precipitation reactions applied to each fraction. Fractionation and solute partitioning - this methodology yields three distinct extractive solutions (fractions), segregating the plant metabolites according to their hydrophobicity and hydrophilicity:

- Fraction A: Ethereal extractive solution contains lipophilic (hydrophobic) compounds.
- Fraction B: Ethanol extractive solution contains amphiphilic and hydrophilic compounds.
- Fraction C: Aqueous extractive solution contains highly hydrophilic compounds.

The qualitative analysis reveals a distinct chemical compartmentalization based on polarity. Fraction A (ether) selectively isolated lipophilic compounds (carotenoids and fatty acids) restricted to the aerial parts (Herba and Flores). Fraction B (ethanol) proved to be the most metabolically rich solvent, consistently extracting the primary polyphenolic classes - flavonoids and polyphenolcarboxylic acids-across all plant organs, indicating a predominance of glycosidic and polar derivatives. The floral organ (Flores) exhibited the highest chemical complexity, being the sole source of anthracenozines, while the root (Radix) was characterized by a strictly hydrophilic profile dominated by tannins, phenolics, and polyuronides.

#### 2.1.4. Root Phytochemical Analysis

**Figure 4:**
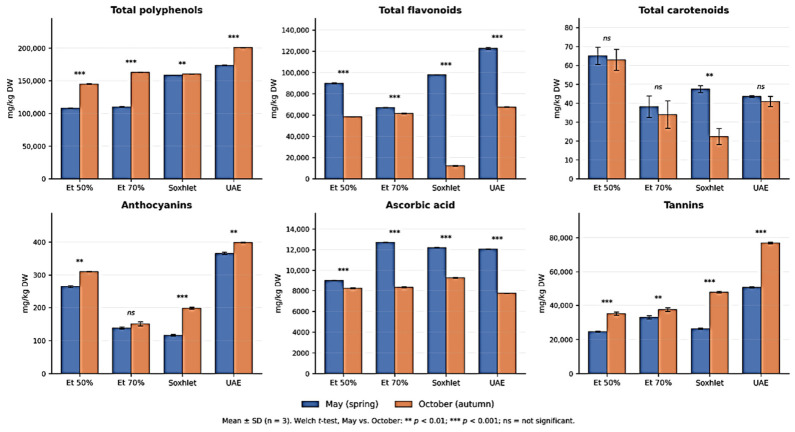
Total polyphenols, flavonoids, carotenoids, anthocyanins, ascorbic acid and tannins content in hydroalcoholic extracts of *Taraxacum* root. Note: Welch *t*-test, May vs. October–mean ± SD (standard deviation), n = 3, ** *p* < 0.01, *** *p* < 0.001, ns = not significant.

Quantitative analysis of bioactive compounds in *Taraxacum* root extracts indicates a distinct hierarchy in secondary metabolite concentrations with flavonoids and tannins constituting the predominant compounds within the root matrix. The concentration of tannins records a maximum in the autumn period, reaching 76,941.33 mg/kg DW in May (Figure 4). Similarly, the total polyphenol content exhibits a significant peak in autumn across all extraction methods, with the highest concentration recorded in the October UAE samples (200,946.42 mg/kg DW, compared to 173,864.17 mg/kg DW in May).

In contrast, secondary metabolites associated with active cell division, antioxidant protection, and an accelerated metabolic rate showed a maximum accumulation during the spring season. The concentration of flavonoids was higher in May, yielding 122,916.51 mg/kg DW compared to 67,581.04 mg/kg DW in October. At the same time, the level of ascorbic acid reached its maximum in the May samples across all analyzed extraction variants. Carotenoids and anthocyanins display consistently low quantitative values compared to the flavonoid fractions (Figure 4). However, their seasonal accumulation tendencies diverged: carotenoid concentrations were higher in the spring (May) samples, whereas anthocyanin levels peaked in the autumn (October) collections.

From a methodological perspective, the results demonstrate that UAE represents the procedure with the highest efficiency in releasing bioactive compounds from the root matrix. This technique generated the highest absolute extraction yields, particularly for the polyphenol (200,946.42 mg/kg DW in October) and flavonoid (122,916.51 mg/kg DW in May) fractions, proving superior to conventional maceration protocols in hydroalcoholic solvents (50:50 (*v*/*v*) and 70:30 (*v*/*v*) ethanol).

The optimization of bioactive compound recovery from *Taraxacum* roots is highly contingent upon both chronobiological and methodological parameters. The integration of an autumn harvest with the UAE constitutes the optimal processing paradigm for maximizing the therapeutic potential of these root extracts.

#### 2.1.5. Leaves Phytochemical Analysis

**Figure 5:**
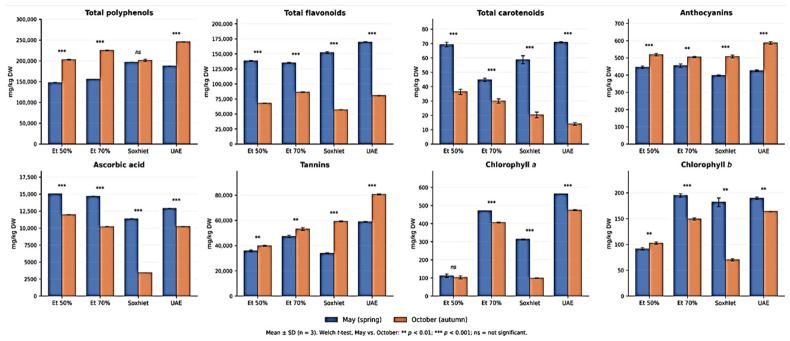
Total polyphenols, flavonoids, carotenoids, anthocyanins, ascorbic acid, tannins, chlorophyll *a*, and chlorophyll *b* content in hydroalcoholic extracts of *Taraxacum* leaves. Note: Welch *t*-test, May vs. October – mean ± SD (standard deviation), n = 3, ** *p* < 0.01, *** *p* < 0.001, ns = not significant.

Phytochemical profiling of the aerial plant parts (Herba) indicates that both solvent polarity and the physical mechanics of extraction modulate the recovery of target components. The Herba matrix is characterized by a substantial presence of photosynthetic pigments (chlorophyll a, chlorophyll b, and carotenoids) concurrent with high concentrations of flavonoids and tannins. Anthocyanin concentrations remained at residual baseline levels ranging from 398.28 to 519.33 mg/kg DW (Figure 5).

The assimilatory pigments reached their maximum quantitative levels during the spring season. For instance, chlorophyll a reached 564.08 mg/kg DW in the UAE, supported by a higher concentration of carotenoids yielding 70.89 mg/kg DW in May, compared to 14.09 mg/kg DW in October. The ratio of chlorophyll a to chlorophyll b based on these spring UAE data points (564.08/189.55 mg/kg DW) was approximately 2.97. Furthermore, ascorbic acid content reached its absolute maximum in May, yielding 12,884.67 mg/kg DW via UAE. The spring period stands out as the maximum metabolic efficiency for *Taraxacum herba*: flavonoid levels were also categorically higher during May, reaching 169,798.66 mg/kg DW via UAE, compared to 80,696.80 mg/kg in October) (Figure 5).

In contrast, the aerial organs exhibited peak concentrations of tannins and total polyphenols during October. Total polyphenol levels peaked consistently in the autumn extracts across all solvent systems and methods, with the highest concentration achieved using UAE in October, yielding an absolute maximum of 245,555.93 mg/kg DW (compared to 187,836.39 mg/kg DW in May). Similarly, UAE generated a tannin yield of 80,708.77 mg/kg DW in autumn, compared to 58,964.93 mg/kg DW in May. Concurrently, a minor increase in the concentration of anthocyanins was observed in the October collections.

The evaluation of the extraction techniques reconfirms UAE as the optimal method for maximizing the recovery of these metabolites. The data clearly highlight the thermal sensitivity of certain compounds: for example, during hot extraction (Soxhlet) in October, the level of ascorbic acid underwent a drastic degradation to 3442.44 mg/kg DW, compared to 10,220.22 mg/kg DW preserved via UAE.

#### 2.1.6. Inflorescences’ Phytochemical Analysis

The analysis of the phytochemical profile of the inflorescences (Flores) of *T. officinale* L. highlights a distinct metabolic specialization compared to the vegetative organs (root and leaves), governed by the reproductive function of the floral capitulum and the interaction with the external environment (entomophilic attraction and photoprotection).

**Figure 6:**
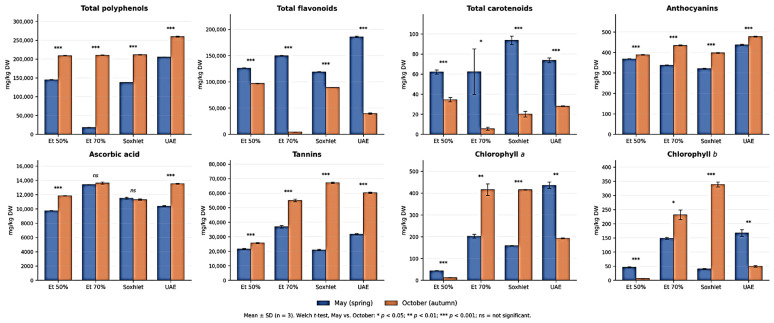
Total polyphenols, flavonoids, carotenoids, anthocyanins, ascorbic acid, tannins, chlorophyll *a*, and chlorophyll *b* content in hydroalcoholic extracts of *Taraxacum* flowers. Note: Welch *t*-test, May vs. October – mean ± SD (standard deviation), n = 3, * *p* < 0.05, ** *p* < 0.01, *** *p* < 0.001, ns = not significant.

Quantitative analysis designates the floral organs as a potent source of flavonoids, with the spring accumulation of both flavonoids and carotenoids consistently recorded across all extraction protocols. Most significantly, under UAE, the flavonoid content surged from 39,667.38 mg/kg DW in October to a peak concentration in May: 185,955.66 mg/kg DW (Figure 6), eclipsing the maximum values observed in both the Radix and Herba matrices. Extracts obtained in May also recorded significantly higher concentrations of carotenoids, yielding 93.70 mg/kg DW via the Soxhlet method compared to 20.13 mg/kg DW in October.

For inflorescences appearing in the autumn period (October), the metabolic profile changed radically. An increase in tannins was recorded, reaching 60,335.29 mg/kg DW in UAE Oct, compared to 31,829.62 mg/kg DW in May. This autumnal surge was mirrored in the total polyphenol content (Figure 6), which consistently peaked in October samples across all tested extraction methods. Specifically, the highest polyphenol recovery was achieved via UAE in October, recording an absolute maximum of 259,651.03 mg/kg DW, representing a significant increase over the spring peak of 205,349.77 mg/kg DW in May.

A notable analytical observation is the quantification of chlorophyll pigments (chlorophyll a and b in the Flores samples. Although the petals (ligules) are non-photosynthetic, the presence of these concentrations (e.g., chlorophyll a of 436.38 mg/kg in UAE Mai) is anatomically explained by the inclusion in the sample of the involucre (green bracts at the base of the capitulum) and the receptacle, which have photosynthetic activity and energetically support flower development, an aspect documented in the morphology of the *Asteraceae* family. Although *T. officinale* L. inflorescences are phenotypically yellow (dominated by carotenoids), the analysis revealed higher concentrations of anthocyanins (e.g., 434 mg/kg DW at 70:30 (*v*/*v*) October). This observation is methodologically justified using hydroalcoholic solvents, which exhaustively extract polar anthocyanins from the capitulum bracts, but underestimate the fraction of lipophilic carotenoids. Also, the presence of anthocyanins confirms the inclusion of the supporting tissues of the flower in the sample, which activate anthocyanin biosynthesis pathways as a mechanism of photoprotection and cold tolerance.

Regarding the extractive yield at the floral level, UAE remained the dominant method for the isolation of most phenolic fractions and labile chlorophyll pigments. However, it is important to highlight that the Soxhlet method generated the best extraction yield for carotenoids in May (93.71 mg/kg DW). Quantitative analysis designates the floral organs as a potent source of flavonoids. The spring accumulation of both flavonoids and carotenoids was recorded across all extraction protocols. Most significantly, under the UAE, flavonoid content surged from 39,667.38 mg/kg DW in October to a peak concentration of 185,955.66 mg/kg DW in May.

#### 2.1.7. Mix Phytochemical Analysis

The phytochemical analysis of a mixed botanical preparation is highly relevant from a pharmacognostic perspective, as traditional phytotherapy frequently utilizes whole-plant (*totum*) extracts to leverage synergistic therapeutic efficacy. The resulting phytochemical profile constitutes a complex matrix that successfully integrates the photosynthetic pigments of the aerial parts, the high flavonoid concentration of the floral organs, and the stable phenolic baseline of the root system.

The seasonal dynamics observed in the individual plant organs are conserved in the mixed extracts; however, peak concentrations are modulated by the amalgamation of distinct tissue matrices. The autumn harvest consistently yielded a significantly richer antioxidant profile. This is reflected in the total polyphenol content, which exhibited a consistent peak in the October samples across all tested methodologies. For example, extraction via 70:30 (*v*/*v*) ethanol yielded a maximum polyphenol concentration of 218,227.92 mg/kg DW in autumn, marking a substantial increase from the 156,149.48 mg/kg DW recorded in May. As observed in the case of isolated organs, autumn remains the optimal season for tannins and anthocyanins extraction. Tannins reach a clear autumnal peak (66,707.38 mg/kg DW in the Soxhlet method and anthocyanins reach 434.62 mg/kg DW in 70:30 (*v*/*v*) cold maceration extraction.

In contrast, the spring integral extract is dominated by metabolites associated with accelerated metabolic rates. Flavonoids reach an absolute maximum during this period (146,641.08 mg/kg by UAE extraction, compared to only 56,133.45 mg/kg in October). The highest levels of ascorbic acid are also recorded in spring, confirming the quality of the whole plant as a source of vitalizing antioxidant agents upon exiting vegetative dormancy.

**Figure 7:**
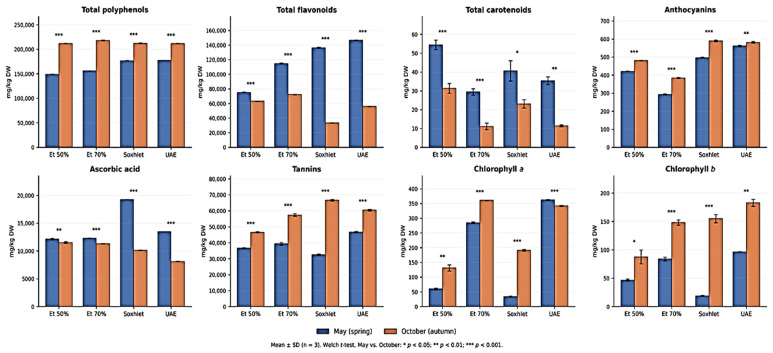
Total polyphenols, flavonoids, carotenoids, anthocyanins, ascorbic acid, tannins, chlorophyll *a*, and chlorophyll *b* content in hydroalcoholic extracts of *Taraxacum* Mix. Note: Welch *t*-test, May vs. October–mean ± SD (standard deviation), n = 3, * *p* < 0.05, ** *p* < 0.01, *** *p* < 0.001.

An analytical peculiarity to highlight is the fraction of pigments (carotenoids and chlorophylls). Compared to the increased chlorophyll values (Figure 7) obtained exclusively from the Herba, the values in the Mix are visibly lower (dilution effect), proving the accuracy of the determinations: the root mass (lacking assimilatory pigments) naturally diluted the total concentration per gram of dry biomass.

The phytochemical results are supported by preliminary physicochemical determinations. The pH values for the Mix samples vary in a slightly acidic range (5.41–6.48), a direct consequence of the abundance of phenolic acids (e.g., chicoric acid, chlorogenic acid) and ascorbic acid in the extracts. Furthermore, the refractive index (%Brix), which measures dissolved solids (including sugars and secondary metabolites), reaches higher values in more energetic extractions (e.g., 23.4 in UAE May and 23.8 in Soxhlet May), reflecting a massive release of solvates.

From an extractive point of view, the UAE and maceration with 70:30 (*v*/*v*) ethanol prove to be the most suitable procedures for the depletion of vegetable *totum*, managing to extract in a balanced way both the water-soluble components and those with a moderate lipophilicity, without subjecting the thermolability of ascorbic acid to prolonged stress (as in Soxhlet extraction).

A notable finding of this study is the quantified presence of ascorbic acid within the mixed extracts, even following conventional Soxhlet extraction, which yielded 19,246.67 mg/kg DW in the May mixture. This observation contributes to the discussion regarding the multi-component profile of whole-plant extracts and their potential role in biochemical interaction. Although the precise mechanisms underlying the stability of thermolabile molecules like Vitamin C during thermal extraction remain to be fully elucidated, these results suggest that the total plant matrix warrants further investigation regarding component interactions.

Similar trends have been discussed in the literature; for instance, ref. [21] hypothesized that a complex biochemical microenvironment—potentially generated by the co-extraction of roots, leaves, and flowers—might introduce various co-extractants, such as structural lipids, pectins, or synergistic polyphenols. It is suggested that these components could act as protective or buffering agents, potentially shielding thermolabile molecules like ascorbic acid from extensive thermal destruction, a phenomenon that requires dedicated compositional analysis to confirm. Furthermore, ref. [22] observed that while isolated tissues (such as Herba) often undergo severe thermal and oxidative degradation of ascorbic acid under stressful conditions, whole-plant mixtures can exhibit a more pronounced “matrix effect”. 

Consistent with the phytochemical ecology of perennial herbs, tannin concentrations in the mixed extracts were significantly elevated during the autumn harvest (e.g., 66,707.38 mg/kg DW via Soxhlet extraction). This value dropped by more than half to 32,506.73 mg/kg DW in the October Soxhlet sample. By autumn, the plant’s metabolic focus shifts toward the accumulation of photoprotective and stress-responsive total polyphenols, an ontogenetic dynamic that is clearly captured and quantified in our whole-plant mixture. The quantitative data confirms this seasonal shift: total polyphenols in the October Mix extracts universally exceeded 211,000 mg/kg DW across all extraction techniques (50:50 (*v*/*v*) ethanol, 70:30 (*v*/*v*) ethanol, Soxhlet, and UAE), overshadowing the spring baseline, which peaked at 177,575.3 mg/kg DW (via UAE).

**Figure 8:**
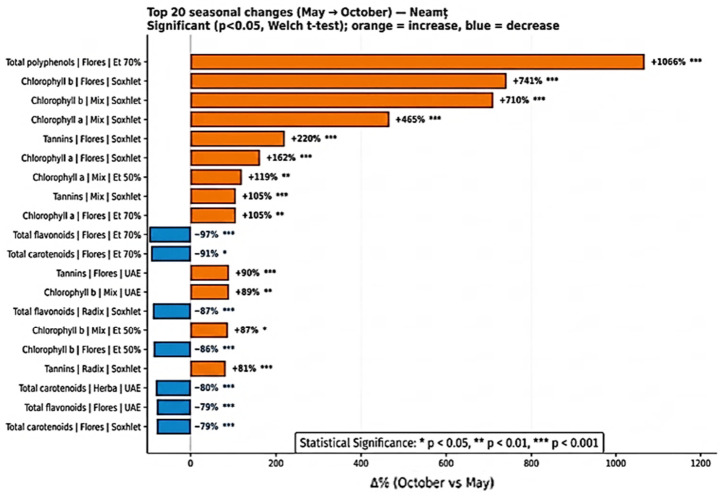
Two-way analysis of variance (ANOVA) of the effects of sampling period and extraction method on the phytochemical parameters of the extracts.

To evaluate the seasonal variation in plant metabolism, the top 20 most significant changes in phytochemical concentrations between May and October for the Neamt samples were analysed using a Welch *t*-test (*p* < 0.05). The resulting data reveal an important seasonal shift in the biochemical profile, characterized by massive accumulations of polyphenols, chlorophylls, and tannins in the autumn, alongside severe depletions of flavonoids and carotenoids. Specifically, the most striking variation overall was observed in the total polyphenols for the Flores extract (70:30 (*v*/*v*) ethanol), which exhibited an extraordinary +1066% increase compared to the May baseline. Concurrently, both chlorophyll *a* and *b* demonstrated substantial autumn accumulation, particularly within the Soxhlet extractions; chlorophyll *b* surged by +741% in the Flores sample and +710% in the Mix sample, while chlorophyll *a* followed a similarly robust upward trend, increasing by +465% in the Mix Soxhlet extract. Tannin concentrations also rose significantly (Figure 8) across multiple plant parts and extraction methodologies, with the highest gain recorded in the Flores Soxhlet extract (+220%), complemented by notable increases in the Mix Soxhlet (+105%) and Radix Soxhlet (+81%) samples.

In stark contrast to these accumulations, several key antioxidant classes experienced statistically significant declines as the vegetative cycle transitioned from spring to autumn. Total flavonoids were depleted by October, with the severe reduction recorded in the Flores 70% extract (−97%), followed closely by the Radix Soxhlet extract (−87%) and the Flores UAE sample (−79%). Total carotenoids plummeted similarly across multiple matrices and extraction methods, with decreases ranging from −79% in the Flores Soxhlet extract down to −91% in the Flores 70% extract. Taken together, these findings demonstrate that the transition from May to October in the Neamt region drives a fundamental biochemical restructuring within the plant tissues, strongly favoring the intense biosynthesis and retention of polyphenolic and tannin compounds while nearly exhausting the baseline flavonoid and carotenoid reserves.

#### 2.1.8. RP-HPLC-DAD Method for Simultaneous Quantification of Phenolic Compounds in Complex Taraxacum Matrices

The qualitative and quantitative determination of polyphenolic profiles from complex plant matrices is an essential step in the quality control, standardization, and bioactivity assessment of phytotherapeutic products. The objective was to advance a sensitive and reproducible protocol capable of achieving simultaneous chromatographic resolution, identification, and quantification of an extensive panel of 23 reference phenolic compounds within a significantly optimized analysis runtime in the UAE samples.

##### RP-HPLC-DAD Radix UAE Phytochemical Profiling

Application of the validated method to the *T*. *officinale* Radix UAE extract resolved multiple peaks, confirming 5 components and revealing 5 tentative or unassigned signals, as detailed in Table 5.

The quantitative results demonstrate that gallic acid is the dominant free phenolic monomer resolved within the root tissues, yielding a high content of 293.716 mg/100 g. The extract also shows an abundant flavonoid fraction, with kaempferol quantified semi-quantitatively at 111.136 mg/100 g. This finding confirms that substantial amounts of highly protective aglycones remain present within the root tissue architecture. The definitive confirmation of free chlorogenic acid and caffeic acid represents the most chromatographically and pharmacologically important finding in the Radix profile, validating the initial analytical interpretations. Chlorogenic acid was positively identified at an observed retention time of 3.536 min, which matches the retention time of the pure reference standard (R_t_ = 3.5350 min) perfectly. This exact match contrasts sharply with the plant’s aerial extracts (*Taraxaci* Herba), where the chlorogenic marker shifts forward to 3.238 min due to the presence of native esterified isomers or leaf-specific caffeoylquinic variants. Free caffeic acid was confirmed at 4.607 min with a calculated content of 1.768 mg/100 g. The prominent presence of these two compounds is consistent with established literature, which reports caffeoylquinic acids, chlorogenic acid, caffeic acid, and related phenolic derivatives as main chemical signatures in both the root and aerial parts of *Taraxacum officinale* L. [23,24]. The hydroxycinnamic acid profile characteristic of high-quality dandelion root materials is further defined by the identification of caftaric acid at 2.158 min, yielding a content of 12.508 mg/100 g. Caftaric acid is designated as a possible match because its peak at 2.158 min was successfully tracked at 310 nm—a wavelength highly suitable and selective for hydroxycinnamic derivatives. The screening also revealed several unassigned peaks at later retention times, specifically at 10.036 min (Figure S2), 11.740 min, and across the 15.451–15.625 min cluster. These resolved fractions exhibit UV spectral characteristics typical of complex hydroxycinnamic or flavonoid derivatives. Their presence suggests that while the UAE effectively isolates target analytes, it also co-extracts a broad array of conjugated compounds from the dense root core.

##### RP-HPLC-DAD Herba UAE Phytochemical Profiling

The optimized RP-HPLC-DAD method successfully resolved the complex botanical matrix of *T. officinale* Herba, distinguishing multiple overlapping peaks within the 22 min gradient window. A total of eight specific phenolic markers were successfully tracked, characterized, and quantified using individual standard spectral matches and calibration functions, as comprehensive in Table 6.

The data indicate that the most chromatographically and therapeutically relevant constituents detected in the *Taraxacum* Herba profile are gallic acid, kaempferol, luteolin (luteol), and cinnamic acid. Quantitative evaluations demonstrate that gallic acid serves as the dominant free phenolic acid, registering a substantial concentration of 287.816 mg/100 g. This high concentration contributes significantly to the extract’s radical scavenging capacity. The flavonoid profile is characterized by a robust concentration of kaempferol (166.138 mg/100 g), balanced by a distinct luteolin fraction quantified at 30.875 mg/100 g. The identification of luteolin is highly meaningful, as the literature widely documents the prominent distribution of luteolin along with various luteolin glycosides specifically within the leaves and flowers of *T. officinale* L. [23,25]. Jointly, these targeted flavonoids provide the physiological foundation for the anti-inflammatory and cellular defence mechanisms associated with dandelion herb preparations. Differentiation was noted regarding the hydroxycinnamic acid derivatives. The prominent peak observed at an early retention time of 3.238 min. (Figure S3) was assigned as a “possible caffeoylquinic derivative” rather than a definitive chlorogenic acid match. This assignment is supported by comparative matrix behaviour: the verified chlorogenic acid signal confirmed in both *Taraxacum* radix (root) and the root/herba/flores mixture consistently elutes at a later window, between approximately 3.536 and 3.553 min. The noticeable negative shift in retention time (R_t_ = 3.238 min) for this herba-specific compound suggests the presence of native esterified configurations, structural isomers, or distinct matrix-induced migration anomalies typical of crude botanical substrates. A secondary signal was resolved at 4.805 min. and classified as a possible caffeic acid or caffeic acid derivative (quantified as 9.905 mg/100 g). The target analytes showed exceptional chromatographic alignment, including minor phenolic acids like syringic acid (19.022 mg/100 g) and p-coumaric acid (6.379 mg/100 g). The clean, simultaneous resolution of early-eluting, highly polar organic structures (such as gallic acid at 0.912 min) alongside late-eluting, strongly hydrophobic flavonoid cores (such as cinnamic acid at 15.739 min) highlights the strong peak separation performance.

##### RP-HPLC-DAD Flores UAE Phytochemical Profiling

The chromatographic separation of the floral matrix revealed a highly concentrated and distinct phenolic signature compared to the previously analysed vegetative and root tissues. A total of 10 primary phenolic signals were tracked, with quantitative and qualitative findings detailed in Table 7.

In the flowers, the absolute highest values for gallic acid and kaempferol occur across all tested segments. Gallic acid was tracked at a massive concentration of 3015.559 mg/100 g, while kaempferol was semi-quantitatively evaluated at 478.287 mg/100 g. However, special analytical attention must be paid here: the initial elution area of the chromatogram (between 0.9 and 1.2 min) is extremely crowded. Consequently, these exceptionally high calculated values may be partially influenced by matrix coelution, where overlapping polar constituents inflate the integrated peak areas.

In addition to these early-eluting compounds, dandelion flowers are extensively documented in scientific literature as being abundant in flavonoids, particularly derivatives of luteolin. This aligns with a recent LC-MS/MS study that successfully quantified 28 distinct flavonoids in flowers and dandelion flower products [3]. Corroborating this, the HPLC screening tracked a specific luteol/luteolin derivative as a possible match at 1.508 min, yielding a semi-quantitative content of 33.799 mg/100 g.

The floral matrix also presents a unique phenolic acid signature. Notably, 3-O-methylgallic acid was strictly confirmed at a retention time of 2.460 min. (118.122 mg/100 g), alongside syringic acid at 4.983 min. (52.573 mg/100 g). Regarding the hydroxycinnamic acids, Flores extract exhibits a possible caffeoylquinic isomer shifted to an earlier retention time of 3.189 min. (22.554 mg/100 g), like the isomer shift observed in the Herba extract.

From a diagnostic perspective, the most important unquantified peak in the floral chromatogram is observed at 16.270 min. (Figure S4). This peak generates a massive integrated area (9416.17773) and presents a very intense signal at 310 nm wavelength. Based on these spectral and chromatographic properties, this may correspond to a major conjugated hydroxycinnamic derivative—possibly cichoric acid, monocaffeoyltartaric/ dicaffeoyltartaric acid, or another higher-order caffeoylquinic derivative. This robust structural assignment is well-supported by established literature, which mentions cichoric acid and monocaffeoyltartaric acid as the main phenolic constituents not only in the flowers but also throughout the roots, leaves, and medicinal preparations of *Taraxacum officinale* [25].

The unique chemical fingerprint confirms the high pharmacological potential of dandelion flowers and emphasizes the need to monitor specific biomarkers, like cichoric acid variants, when standardizing floral-derived phytomedicines.

##### RP-HPLC-DAD Mix UAE Phytochemical Profiling

The resulting chromatographic profiles revealed significant organ-specific variations and highlighted the complex chemical synergy captured when pooling different morphological structures. The quantitative and qualitative datasets for both matrices are compiled in Table 8.

The HPLC-DAD reports for the Mix sample provide clear, reliable resolution at the 310 nm monitoring wavelength, which is highly selective for hydroxycinnamic acid skeletons. At 310 nm wavelength, the profile shows distinct signals for key therapeutic targets: true chlorogenic acid is confirmed at 3.553 min. (56.904 mg/100 g), free caffeic acid resolves cleanly at 4.625 min. (33.398 mg/100 g), and p-coumaric acid appears at 7.161 min (15.298 mg/100 g). Additionally, a substantial peak at 2.147 min. is assigned as a possible match for caftaric acid, yielding an accumulated content of 124.347 mg/100 g. The Mix extract also shows the late-eluting major hydroxycinnamic derivative at 16.262 min. (Figure S5). However, its integrated peak area (1062.16956) is noticeably lower than the massive peak seen in the pure floral extract, illustrating a clear dilution effect caused by mixing the flowers with the fibrous root and leaf tissues. By integrating the distinct chemical profiles of the roots, leaves, and flowers, the Mix extract provides a well-rounded polyphenol profile that fits standard quality control frameworks for comprehensive *T. officinale* L. monographs.

### 2.2. Antimicrobial Activity

The results indicate a selective antimicrobial activity of dandelion extracts, with better efficiency especially against Gram-positive bacteria (*Staphylococcus aureus* ATCC 25923, *Enterococcus faecalis* ATCC 29212) and low or absent activity in many cases against Gram-negative bacteria (*Escherichia coli* ATCC 25922, *Pseudomonas aeruginosa* ATCC 27853) and against *Candida albicans* ATCC 10231 compared to the solvent used (50:50 (*v*/*v*) ethanol or 70:30 (*v*/*v*) ethanol) (Figure S6).

In the case of Gram-positive bacteria, inhibition zones frequently appear with diameters ranging between 7.00 and 16.00 mm, accompanied by moderate MIC value ranges (500–62.5 µL-mL). These parameters correspond to the direct utilization of the undiluted stock extracts, which had quantified dry residue concentrations ranging from 15.2 to 25.2 mg/mL across all variations. Specifically, the baseline dry matter yields varied according to the specific organ matrix and the extraction methodology employed, establishing specific ranges for each tissue: 16.8–25.2 mg/mL for Radix, 15.2–22.6 mg/mL for Herba, 16.2–23.4 mg/mL for Flores, and 15.8–23.8 mg/mL for the combined Mix preparations. Within the dataset, the MIC values of the extracts that performed better than the standalone solvent controls are highlighted in yellow (Table 9). Conversely, for most of the Gram-negative bacteria and the yeast strains, several extracts produced IZD of 0 mm; consequently, quantitative secondary evaluations—including MIC, MMC, and MICMA were not performed for these combinations. The tested extracts demonstrated an absolute absence of qualitative antifungal effects against *Candida albicans*, generating an IZD of 0.00 mm across all sample variations.

The semi-quantitative parameter evaluating the MICMA was typically equal to or less than the standard MIC, a result of the biofilm’s mechanical and metabolic defenses. In Table 9, the variants presenting an MICMA value lower than the MIC are highlighted in blue. This anti-adherence profile was narrow and primarily limited to Gram-positive bacteria, where the MICMA was relatively low (62.5–125 µL/mL), though other samples exhibited high MICMA values. A key exception was noted for the Flores UAE extract, which displayed the most pronounced anti-adherence profile against Gram-negative bacterial cells. Dandelion extracts showed selective antibacterial action, primarily against Gram-positive bacteria (*S. aureus*, *E. faecalis*), with IZDs of up to 16 mm and MICs ranging from 62.5 µL/mL to 500 µL/mL, depending on the microbial strain. Although there were some noteworthy exceptions, such as *E. coli* being inhibited by Flores UAE and *P. aeruginosa* being inhibited by Mix 70%, and some extracts obtained through Soxhlet and UAE methods, Gram-negative bacteria were generally more resistant.

The extracts in ethanol 70:30 (*v*/*v*) had, overall, better performance than those in ethanol 50:50 (*v*/*v*), indicating a more efficient extraction of bioactive compounds. The UAE generated the largest inhibition zones on Gram-positive bacteria for Herba and Mix, suggesting an advantageous method for obtaining extracts with antimicrobial potential.

The *in vitro* in vitro antimicrobial assessment of the May extracts (Table 10) reveals a unique pharmacological profile compared to the autumnal harvest. While the spring extracts demonstrate notable selective antibacterial activity against Gram-positive pathogens and specific Gram-negative strains, they consistently lack antimycotic effectiveness against *C. albicans,* as indicated by an IZD of 0.00 mm across many sample variations.

The most potent antimicrobial activity within the May harvest was recorded against the Gram-positive strains. The Mix UAE and Herba UAE samples exhibited the maximum IZD, reaching 16.00 mm and 15.33 mm against *E. faecalis*, respectively, and 15.00 mm against *S. aureus.* Quantitative comparison of the MMC relative to the MIC demonstrated that the MMC was frequently equal to the MIC or 2x ×MIC, and in some cases even >500 µL/mL, meaning that the bactericidal level was not reached within the tested range. The extracts prepared in 50:50 (*v*/*v*) ethanol were predominantly bacteriostatic. However, the 70:30 (*v*/*v*) Mix macerate exhibited a direct bactericidal effect against its target, with an MIC of 125 µL/mL and an MMC of 250 µL/mL (where MMC = MIC relative to the established thresholds). Notably, a matching MIC and MMC value was recorded for *E. faecalis* across multiple variants: Herba 50%, Mix 50%, Mix Sox, Flores Sox, and Flores UAE.

The extracts prepared in 50:50 (*v*/*v*) ethanol seem to extract active compounds sufficiently well against Gram-positive bacteria, but generally not enough to overcome the barriers of Gram-negative ones. Moreover, the 50:50 (*v*/*v*) flower extracts have lower IZD than Herba/Mix, suggesting either a lower concentration of relevant active compounds or a completely different phytochemical profile.

The most potent antimicrobial activity within the May harvest was recorded against Gram-positive bacteria. Strong bactericidal and bacteriostatic effects were observed against *S. aureus* and *E. faecalis*, particularly in the Herba and Mix samples.

Although Gram-negative strains (*P. aeruginosa* and *E. coli*) demonstrated higher overall resistance, specific extraction parameters yielded broader-spectrum activity. The Flores UAE extract maintained a notable inhibitory effect, recording an IZD of 12.00 mm for both *P. aeruginosa* and *E. coli*, alongside a low MIC value of 62.5 µL/mL for *P. aeruginosa*. Additionally, the Herba 70% sample demonstrated moderate inhibitory activity against *E. coli* (IZD of 8.00 mm).

In terms of methodology, the 70:30 (*v*/*v*) ethanol generally outperformed those in 50:50 (*v*/*v*) ethanol. For example, Herba 70% achieved a 15.00 mm IZD against *S. aureus*, compared to 10.00 mm for Herba 50%. Furthermore, the UAE method consistently generated broader-spectrum antimicrobial activity and larger inhibition zones compared to the Soxhlet counterparts.

To evaluate the key factors influencing the antimicrobial activity of the extracts, non-parametric statistical analyses were conducted. The results (Table 11) demonstrated a significant variation in susceptibility across the five tested microbial strains (H = 231.84, *p* < 0.001), indicating that the efficacy of the extracts is highly strain-dependent.

Furthermore, a Mann–Whitney test revealed a significant difference in sensitivity between Gram-positive and Gram-negative bacteria (U = 21.242, *p* < 0.001). As observed in our biological assays, Gram-positive strains exhibited greater overall susceptibility compared to Gram-negative strains. This difference is likely attributable to the structural variations in their respective cell walls, particularly the presence of the protective outer membrane in Gram-negative species.

Interestingly, a Kruskal–Wallis analysis assessing the impact of the extraction method showed no statistically significant difference in antimicrobial outcomes (H = 6.57, *p* = 0.087). This indicates that while the extracts possess strong, targeted antimicrobial properties depending on the bacteria they face, the specific method used to extract the plant material did not fundamentally alter their overall antimicrobial potency.

### 2.3. In Vivo BSLA (Brine Shrimp Lethality Assay)

**Figure 9:**
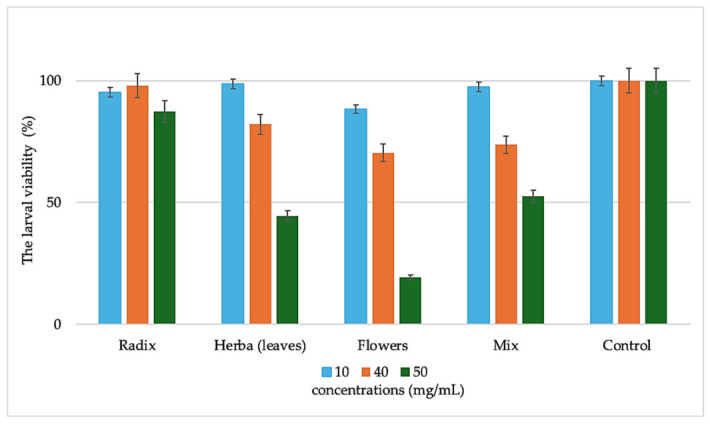
Larvae viability (%) ± SD after 96 h exposure at different concentrations of extracts: 10 mg/mL, 40 mg/mL, and 50 mg/mL.

Four ethanol extracts of *T. officinale* L. obtained by UAE: Radix, Herba, Flores, and Mix were tested. In all experimental tanks, viability was high (over 80%) at concentrations of 1–10 mg/mL for all categories of extracts. Periodic evaluation allowed the identification of a decrease in swimming capacity and its limitation to the bottom of the experimental tanks, in specimens exposed to Herba and Flores extracts after 48 h from the beginning of the experiment. Optika Italy, B-350 microscope, was used to observe details of the bio tester organisms. Also, tremor-like movements of the appendages used for swimming (antenna 2) were noted, which can be explained by the interaction of the composition of the extracts with neuronal action. Lethality in the test population (Figure 9) is preceded by distinct neuromuscular dysfunctions, characterized by the disruption of rhythmic antennary movement and a loss of systemic coordination.

These ethological aberrations manifest as intermittent, spasmodic, and desynchronized motor activities, which indicate an underlying neurological interference. From an ecotoxicological standpoint, the recorded mortality at extract concentrations higher than 1000 µg/mL (LC_50_) suggests non-toxic activity that supports applications in phytotherapy for the studied plant (Table 12).

#### 2.3.1. Daily Relative Growth Rate (RGLD)

**Figure 10:**
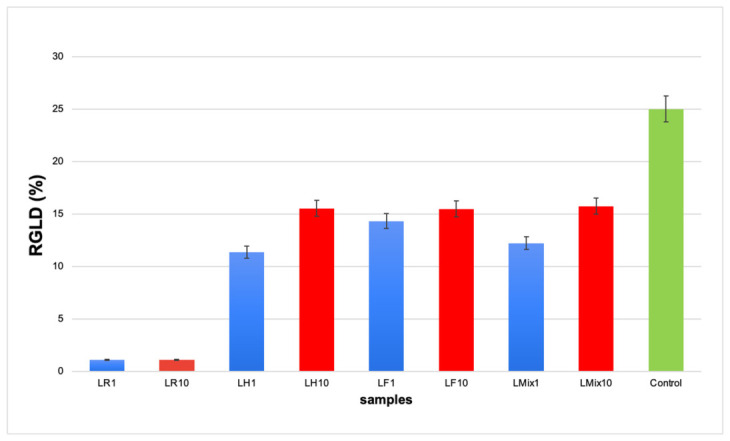
Daily relative growth larvae rate (RGLD) by analysing the average lengths of larvae exposed to concentrations of 1 mg/mL (blue), compared to those exposed to concentrations of 10 mg/mL (red); LR = larvae in root extract, LH = larvae in Herb/leaves extract, LF =larvae in flowers extract; LMix = larvae in mixture of extracts (R + H + F) (±SE).

The growth of larvae is significantly influenced by the type of extract and confirms the results (Figure 10). The specimens exposed to the root extract show the lowest RGLD (2%), while exposure to the other samples induces a growth rate between 11.34% (LH1) and 15.75% (LMix10). RGLD does not show significant changes correlated with the extracted concentration value that was used for the analysis. Thus, the samples LH10, LF10, and LMix10 show small differences of 1.15% (LF10), 3.54% (LMix10), and 4.119% (LH10) compared to LF1, LMix1, and LH1, respectively. These observations can be correlated with the fact that the larvae are different from each other in that they hatch at intervals of a few hours, which for this biological system means growth from one larval stage to another, and, respectively, growth in length.

Observations regarding larval ontogeny indicate that the digestive tract becomes functional approximately 24 h post-hatching, facilitating ingestion of filtered organic matter, including botanical extracts. It was hypothesized that a tenfold increase in extract concentration (10×) would result in either exacerbated cytotoxicity or accelerated morphogenesis; however, these hypotheses remain experimentally unverified. Comparative analysis of larval length against the control group reveals significant phenotypic disparities, suggesting that all tested extracts induced a systemic inhibition of cellular proliferation and differentiation.

#### 2.3.2. Statistical Analysis

Comparative analysis between the experimental groups, regarding the size of the larvae, highlights a significant statistical difference (Tukey B) (*p*-value < 0.005) for all samples vs. control samples. Also, high statistical significance appears (Table 13) between the size of the specimens maintained in Radix extract and the size of the larvae from the other samples (Herba, Flores, Mix).

#### 2.3.3. PCA

**Figure 11:**
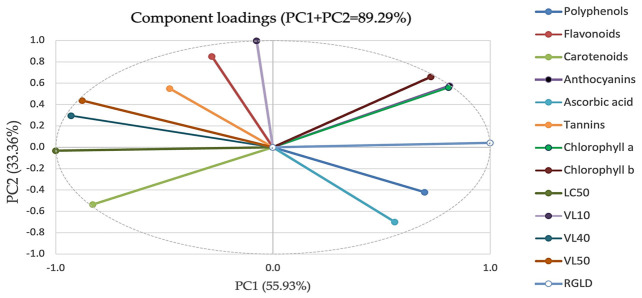
*Anova* test—Principal component analysis of variables analyzed at *Taraxacum* extracts—LC_50_—lethal concentration for 50% of tested organisms; VL10—larval viability at a concentration of 10 mg/mL; VL40—larval viability at a concentration of 40 mg/mL; VL50—larval viability at a concentration of 50 mg/mL; RGLD—daily larvae growth rate (%).

Principal Component Analysis (PCA—Figure 11) reveals that the first two components (PC1 and PC2) account for 89.29% of the total variance, indicating a robust data structure. An analysis of the component loadings for the two components, PC1 and PC2, reveals a strong positive correlation (>0.50) between phytochemicals such as polyphenols, chlorophyll a and b, and the larval development rate. Furthermore, these same chemical compounds negatively influence larval viability at high concentrations (VL40 and VL50) as well as the toxicity indicator LC_50_. Component PC2 groups variables with a significant correlation between the increase in phytochemicals—polyphenols, anthocyanins, and tannins—and larval viability at the lowest concentration tested (VL10).

**Figure 12:**
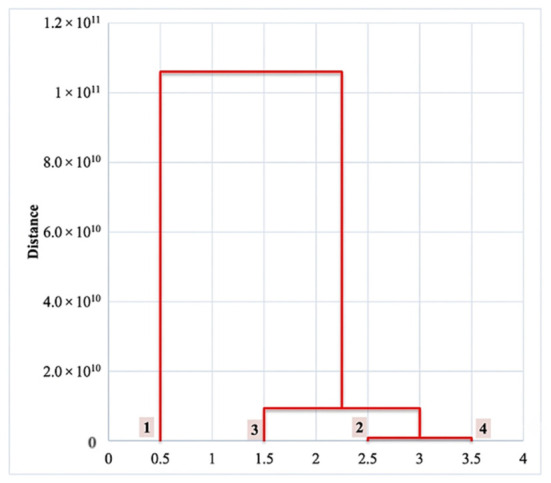
Hierarchical cluster analysis dendrogram. Note: line 1 = Radix; line 2 = Herba, line 3 = Flores; line 4 = Mix.

Hierarchical cluster analysis, visualized via the dendrogram (Figure 12), identifies three distinct groupings based on integrated phytochemical profiles and induced biological effects. The first cluster is uniquely defined by the Radix (line 1) treatments, while the second is characterized by data associated with the floral extract (line 3). The third cluster demonstrates a high degree of similarity between the Herba (line 2) and Mix (line 4) formulations. These taxonomic associations demonstrate significant congruence with the experimental outcomes observed in both larval viability assays and RGLD assessments. Such clustering suggests that the specific secondary metabolite composition of each plant part is the primary driver of the observed phenotypic responses in the larval model.

## 3. Discussions

The comprehensive characterization of *T. officinale* L. extracts reveals an intricate interplay between biological chronobiology, anatomical specialization, and technological extraction mechanics. Plant secondary metabolism is fundamentally dynamic rather than static; the quantitative shifts documented across the Radix, Herba, and Flores matrices represent highly evolved physiological adaptations to changing seasonal stressors. During spring, cellular assets are structurally directed toward active organogenesis, reproductive signalling, and photosynthetic optimization, driving up the production of flavonoids, light-harvesting assimilatory pigments, and mobile organic antioxidants. Conversely, the transition into autumn triggers a systemic resource redirection, shifting the physiological focus toward parenchymal tissue preservation, cold tolerance, and subterranean microbial defences, which manifests as an overexpression of complex polyphenols and condensed tannins.

### 3.1. Evaluation of the Physicochemical Analysis of Vegetal Organs

The seasonal and anatomical fluctuations in the physicochemical parameters of *Taraxacum officinale* L. extracts are deeply rooted in the vegetative lifecycle of the species and the chemical laws governing solid–liquid extraction.

The significant elevation of %Brix values documented in the October root extracts is driven by the late-season physiological redirection of nutrients. During the autumnal transition, the plant actively translocates metabolic assets from the senescing foliar apparatus down into the subterranean storage tissues, accumulating dense concentrations of reserve carbohydrates, most notably the fructan inulin [26]. The concurrently higher acidity observed in this autumn roots suggests a parallel seasonal fluctuation in the synthesis and vacuolar storage of organic acids within the root cortex [27].

Conversely, the elevated electrical conductivity values observed across all May samples reflect the high presence of dissolved mineral salts and mobile electrolytes. This phenomenon directly correlates with the rapid vegetative growth phase characteristic of spring, during which the root system maximizes the active absorption of water and inorganic minerals from the soil to logistically support the development of newly forming aerial tissues [28]. The fact that Herba extracts consistently displayed higher conductivity levels than Radix matrices under identical conditions further confirms the targeted translocation of these inorganic ions to the foliar apparatus, where they serve as essential cofactors in the photosynthetic machinery [29].

However, the rapid degradation of these foliar tissues during late autumn explains the sharp drop in soluble solids (22.2% down to 16.2% Brix) observed in the autumn Herba UAE extracts. As the plant undergoes senescence to prepare for winter dormancy, foliar metabolites are either translocated or enzymatically broken down, leading to a depleted leaf matrix [30]. A similar senescent limitation explains why spring flowers outperformed secondary October blooms under advanced extraction protocols; May inflorescences develop under optimal physiological conditions characterized by peak nectar yields and maximum flavonoid synthesis for pollinator attraction [23,31], whereas late-season blooms emerge under severe abiotic stress, yielding lower concentrations of easily extractable metabolites. The UAE for the May flowers achieved a similarly high yield (22.4% Brix) while preserving a more neutral pH (5.81), proving that acoustic cavitation is a superior, non-destructive method for extracting delicate floral tissues. Furthermore, early-season (May) flowers responded much better to advanced extraction techniques than late-season (October) secondary blooms. The UAE method extracted significantly more soluble solids from May flowers (22.4% Brix) compared to October (15.8% Brix). Spring flowers, blooming under optimal conditions, possess maximum nectar yields and peak flavonoid synthesis for pollinator attraction [22,31]. Whereas autumnal flowers developing under environmental stress exhibit lower concentrations of easily extractable soluble metabolites.

The distinct behaviours observed in the floral matrices when shifting solvent polarities are fully grounded in the chemical principles of dielectric constants. Because pure ethanol possesses a substantially lower dielectric constant than water, elevating the solvent concentration from 50:50 (*v*/*v*) to 70:30 (*v*/*v*) reduces the overall polarity of the hydroalcoholic mixture. This modification renders the solvent less effective at dissolving highly polar inorganic mineral salts (explaining the drop in electrical conductivity), yet highly efficient at penetrating cellular boundaries to solubilize the moderately polar phenolic acids and luteolin glycosides that dominate the floral profile [4,32,33]. 

Continuous, high-temperature exposure induces the thermal degradation of thermolabile floral components, generating acidic thermal artifacts and degradation byproducts. The continuous thermal reflux of the Soxhlet method and the acoustic cavitation generated by UAE both successfully disrupt rigid plant cell walls to maximize intracellular release [33,34]. However, the severe drop in pH (to 5.01) observed during the floral Soxhlet extraction highlights the vulnerability of the delicate floral matrix. Conversely, UAE achieves comparable extraction efficiencies under mild thermal conditions, utilizing acoustic shockwaves to safely isolate the phytocomplex without inducing degradation.

Finally, the whole-plant Mix extracts demonstrate the critical role of matrix interactions and biomass distribution. The profound buffering effect observed in the Mix Soxhlet extract (pH 6.48 vs. the isolated root pH 5.00) indicates that the integration of aerial parts introduces basic mineral salts and alternative organic buffer systems that effectively neutralize the highly acidic resins and fractions isolated from the root matrix [35]. Furthermore, the complete inversion of the seasonal %Brix trend in the Mix sample—where spring extracts outperformed autumn extracts—reflects shifting ratios of biomass. In May, the plant possesses an abundance of highly active, succulent aerial biomass rich in easily extractable sugars, vegetative proteins, and mucilage. In autumn, the senescing foliar tissues lose these mobile fractions, acting as a low-yield diluent that suppresses the overall extraction efficiency of the dense root mass within a combined sample framework [36,37].

### 3.2. Seasonal Fluctuations in Essential Micronutrients and Ecotoxicological Implications

The pronounced mobilization of iron (Fe) observed in May (3.89–7.67 mg in roots) aligns with the findings of [38], who characterized *T. officinale* L. as a highly efficient mineral accumulator during its peak vegetative growth phase. This vernal surge satisfies the heightened metabolic demand for Fe, an essential cofactor in the enzymatic biosynthesis of chlorophyll a and b. As identified by [21], the transition from winter dormancy to active flowering triggers a robust source-to-sink translocation of Fe to aerial tissues (Herba), accounting for the nearly fourfold concentration increase (1.76–6.62 mg/L) documented in this study. Conversely, the marked accumulation of zinc (Zn) within the root architecture during October (2.50 mg/L) and its subsequent depletion in spring (0.32 mg/L) exemplifies a classic seasonal nutrient translocation strategy. Perennial ruderal species leverage their taproots as metabolic reservoirs to sequester divalent cations during autumn senescence, a phenomenon corroborated by [39]. This sequestered Zn reservoir is vital for the synthesis of endogenous auxins (growth hormones) required to drive rapid spring budburst and shoot elongation (see Figure 1). Autumn represents a period of pronounced organic matter turnover driven by foliar senescence and the decay of ephemeral annual root systems. As the soil microbiota community decomposes this fresh detritus, it releases an influx of low-molecular-weight organic acids (LMWOAs), principally citric and malic acids, into the rhizosphere [40]. These LMWOAs function as highly effective natural chelating agents; by forming stable complexes with insoluble, soil-bound zinc fractions, they significantly reduce rhizosphere pH and displace Zn from adsorption sites, converting it into water-soluble and highly bioavailable Zn^2+^ forms ready for plant uptake [41]. Concurrently, the escalation of copper (Cu) in floral organs from October to May (0.07–0.11 mg/L) reflects the upregulation of antioxidant defence systems characteristic of flowering angiosperms [42]. Manganese (Mn) and Cu serve as structural and catalytic components of superoxide dismutase metalloenzymes (Mn-SOD and Cu/Zn-SOD). In response to increasing solar radiation and photoperiod in May, *T. officinale* L. upregulates these microelements within reproductive tissues (Flores) to mitigate ultraviolet (UV)-induced oxidative stress and safeguard reproductive fitness.

The negligible concentrations of lead (Pb) and cadmium (Cd) falling below the limit of detection (<LOD) carry profound ecotoxicological implications. Given that *T. officinale* is a validated hyperaccumulator and bioindicator for heavy metal contamination [43], the absence of detectable xenobiotic metals provides a reliable empirical proxy confirming the environmental purity of the harvest site, thereby satisfying the stringent safety thresholds mandated for pharmaceutical-grade botanical extracts.

### 3.3. Seasonal Fluctuations in Spatiotemporal Allocation of Secondary Metabolites

The seasonal variations observed in the metabolic profile of *Taraxacum* roots reflect distinct physiological adaptations across the perennial vegetative cycle. The elevated levels of tannins and total polyphenols documented during autumn are consistent with literature emphasizing the essential role of these compounds in providing a chemical defence barrier for the root system against pathogenic, microbial, and fungal agents in the soil during the cold season [44]. 

The empirical data demonstrates that the anatomical architecture of *T. officinale* L. dictates its localized chemical profile. This observation aligns with established botanical paradigms showing that secondary metabolites are distributed heterogeneously across plant tissues based on specialized biological functions, such as defence, reproduction, and photosynthesis [44,45]. This qualitative and quantitative phytochemical expression is highly contingent upon ontogenetic stages, anatomical distribution, and the thermo-dynamic parameters of the chosen extraction methodology.

Conversely, secondary metabolites associated with active cell division, antioxidant protection, and accelerated metabolic rates peak during the spring. Although the root system lacks photosynthetic capacity, the onset of the spring vegetative cycle involves a massive systemic biosynthesis of flavonoids, a fraction of which is translocated to the root to support organogenesis and growth processes [46]. The maximum levels of ascorbic acid observed in May further confirm the metabolic consumption or oxidative degradation of vitamin C during the warm summer and autumn months, followed by a resynthesis that peaks at the end of winter dormancy to initiate intense cell division.

The low levels of carotenoids and anthocyanins recorded in the roots align with established botanical principles; because the subterranean matrix is not exposed to sunlight, it does not require UV protection or functional photosynthetic pigments. The slight inflation of these values during the spring likely reflects the transit of synthesized metabolites through the vascular system toward newly forming aerial structures [32].

The quantitative parameters obtained from the *Taraxaci herba* matrix align with current literature, substantiating both the quality of the indigenous biomass and the analytical methodologies applied. The physiological state of accelerated growth during the spring is confirmed by elevated levels of assimilatory pigments. The calculated chlorophyll a to chlorophyll b ratio of approximately 3:1 confirms an optimal photosynthetic efficiency of the harvested biomass, representing the established physiological standard for plants adapted and exposed to direct light [26,47]. Moreover, the absolute maximum concentration of ascorbic acid in May (15,035.77 mg/kg DW) validates the essential role of vitamin C within the active antioxidant network of the foliar apparatus during periods of high metabolic activity. The spring period stands out as the phase of maximum metabolic efficiency for the aerial organs, characterized by a massive accumulation of flavonoids. The upregulated biosynthesis of flavonoids—such as luteolin and its glycosylated derivatives—in young leaves is essential for shielding vulnerable tissues from UV radiation and neutralizing reactive oxygen species (ROS) generated during intense photosynthesis [48].

Conversely, the autumnal overexpression of tannins (80,708.77 mg/kg DW) and total polyphenols in the leaves serves as a typical biochemical marker of foliar senescence. As the plant prepares for winter dormancy, declining leaves synthesize phenolic acids and tannins to protect the remaining tissues against the oxidative and biotic stressors generated by dropping temperatures. This late-season transition is further supported by the observed increase in anthocyanin concentrations in October (peaking at 519.33 mg/kg DW in Herba), a phenomenon that provides compensatory photoprotection to the vascular structure as the principal chlorophyll pigments undergo degradation [49,50].

The dynamic variations observed during the spring period reflect a programmed allocation of secondary metabolites to support reproduction and defence. The intense yellow colour of dandelion inflorescences is primarily determined by the accumulation of lutein and its epoxide derivatives, which play a crucial role in attracting pollinating insects during the period of maximum reproductive receptivity [21]. Simultaneously, the maximum expression of flavonoids observed in May indicates a synergistic co-pigmentation phenomenon with carotenoids. Present within the petals, these flavonoids—especially luteolin and apigenin derivatives—ensure effective shielding of the reproductive organs against intense ultraviolet radiation during spring and early summer [25,47]. These quantitative findings fall well within the established natural physiological parameters for *Taraxacum* species. Comparable studies by [51,52] reported total carotenoid concentrations ranging between 6.34 and 13.44 mg/100 g DW in dandelion organs. Furthermore, the peak flavonoid values strongly corroborate prior literature reporting a total flavonoid content of 188.84 mg/100 g DW in dried *T. officinale* L. samples [53]. These elevated concentrations confirm that dandelion inflorescences act as major physiological sinks for flavonoids, which are synthesized to provide structural photoprotection and pigmentation [54]. Conversely, the metabolic profile of late-season inflorescences shifts radically. The inversion of the phytochemical ratio in October demonstrates that, under conditions of decreasing temperatures and increased abiotic stress, the resources of late inflorescences are redirected from pollinator attraction to the synthesis of antifungal and antimicrobial defence metabolites characteristic of senescence processes [55,56].

The presence of chlorophyll pigments and anthocyanins in the phenotypically yellow Flores samples is justified by anatomical and analytical parameters. Although the petals (ligules) are non-photosynthetic, the inclusion of the involucre (green bracts at the base of the capitulum) and the receptacle accounts for the chlorophyll content, as these supporting tissues possess photosynthetic activity to energetically support flower development. Methodologically, the use of hydroalcoholic solvents exhaustively extracts polar anthocyanins from these capitulum bracts, while potentially underestimating the lipophilic carotenoid fraction. The presence of anthocyanins further confirms the inclusion of these supporting structures, which activate anthocyanin pathways as a mechanism of photoprotection and cold tolerance.

The choice of extraction methodology introduces a critical thermodynamic variable to the phytochemical yield. Methodologically, the distinct superiority of UAE over conventional hydroalcoholic maceration can be attributed to the acoustic cavitation phenomenon. The mechanical effects of cavitation enhance solvent penetration into the dense plant matrix, disrupt cell walls, and promote the rapid release of intracellular components into the medium [57]. Consequently, the optimization of bioactive compound recovery from *Taraxacum* roots is highly contingent upon both chronobiological and technological parameters, demonstrating that the integration of an autumn harvest with UAE processing constitutes the optimal paradigm for maximizing the therapeutic potential of these root extracts. From a technical perspective, the performance of the working techniques reconfirms UAE as the superior method for the valorization of aerial biomass, ensuring enhanced recovery of labile metabolites. The mechanical impact of acoustic cavitation avoids the severe thermal degradation observed during continuous hot extraction (Soxhlet). The sharp drop in ascorbic acid content during Soxhlet processing underscores the inadequacy of prolonged thermal methods for extracting thermosensitive fractions from *Taraxaci herba*, matching established kinetic models regarding the thermal degradation of plant-derived bioactive compounds [42]. Specifically, in May-harvested Herba, ascorbic acid dropped from 15,035.77 to 11,355.76 mg/kg DW under Soxhlet conditions. The superior extraction yield of carotenoids via the Soxhlet method in May (93.70 mg/kg DW)—contrasting with UAE’s dominance for other phenolics—is driven by the specific chemical properties of the target pigments. Due to the strong lipophilic character and relative thermostability of carotenoids, they are efficiently solubilized by the continuous hot reflux of the solvent, which facilitates their release from the dense chromoplasts of the floral cells.

Comparative phytotherapeutic profile of isolated organs vs. the *totum* (Mix). A comprehensive analysis across individual plant organs and the mixed botanical preparation (*totum*) reveals a distinct chronobiological shift in secondary metabolite allocation. The obtained data reveal that the spring harvest represents the peak of metabolic activity, specifically for flavonoids and ascorbic acid, driven by the requirements of active growth and UV photoprotection. During this period, the flowers emerge as the most potent source of flavonoids, reaching a maximum concentration of 185,955.55 mg/kg DW, while the aerial parts provide a superior yield of ascorbic acid (15,035.77 mg/kg DW). This high-energy state is further supported by elevated levels of chlorophyll a (564.08 mg/kg DW in Herba) and carotenoids (93.70 mg/kg DW in Flores), which function as essential light-harvesting and protective pigments. In contrast, the autumn profile reflects a strategic shift toward defence and tissue preservation. The concentration of tannins increases significantly across all organs as the plant prepares for winter, peaking at 76,941.33 mg/kg DW in roots and at 80,708.77 mg/kg DW in the aerial parts. This seasonal surge in tannins provides a robust astringent and antimicrobial barrier, essential for protecting the storage organs during dormancy. Simultaneously, the increase in anthocyanins in October serves as a compensatory photoprotective mechanism as chlorophyll levels decline. The mixed extract (Mix) offers a stabilized biochemical profile that mitigates the extreme seasonal and anatomical fluctuations observed in isolated organs. By maintaining significant levels of flavonoids in the spring (146,641.14 mg/kg DW) and tannins in the autumn (66,707.38 mg/kg DW), the *Taraxaci* Radix, Flores et Herba preparation ensures a comprehensive synergistic effect, making it the most versatile candidate for multi-targeted phytotherapeutic applications.

**Figure 13:**
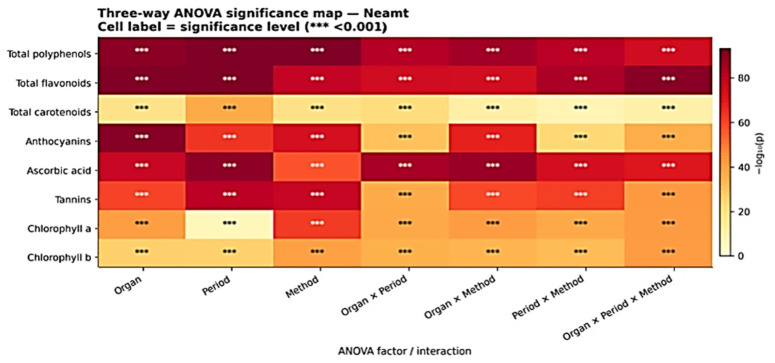
Three-way ANOVA significance heatmap evaluating the effects of plant organ, harvesting period, and extraction method on the phytochemical profile of *Taraxacum officinale* from Neamt region, Romania.

The heatmap (Figure 13) illustrates the statistical significance of three main independent factors (organ, period, and method) and their two-way and three-way interactions across eight dependent variables: total polyphenols, total flavonoids, total carotenoids, anthocyanins, ascorbic acid, tannins, chlorophyll a, and chlorophyll b. The colour gradient corresponds to the −log_10_(*p*) values, where darker red hues denote a higher degree of statistical significance. As shown, all evaluated main effects and their interactions are highly significant (*p* < 0.001) for all tested phytochemical parameters.

The efficiency of plant cell wall disruption and subsequent solubilization of targeted *Taraxacum* phytocompounds is fundamentally dictated by the selected extraction methodology and thermodynamic parameters. Documented literature highlights that UAE provides exceptional extraction efficiency due to acoustic cavitation. The propagation of high-frequency sound waves through the solvent matrix initiates alternating compression and rarefaction cycles, creating microscopic bubbles that rapidly implode. This violent collapse generates intense localized mechanical shear stress that completely ruptures the cellular walls of the plant matrix, significantly accelerating the mass transfer of intracellular components—such as flavonoids and phenolic acids—into the surrounding solvent [57]. Concurrently, the degree of analyte solubilization is strictly governed by the polarity of the extraction phase. Optimization studies on *T. officinale* L. confirm that varying the ethanol-to-water ratio (e.g., 50:50 *v*/*v* vs. 70:30 *v*/*v*) selectively targets distinct compound classes. Specific binary hydroalcoholic mixtures maximize the recovery of both hydrophilic and moderate lipophilic antioxidants by matching the dielectric constant of the solvent matrix to the target metabolites [58]. The experimental data demonstrate that the organic solvent percentage drastically alters quantitative yields. For example, within the spring vegetative matrix (Herba May), 70:30 (*v*/*v*) extracts yielded significantly higher chlorophyll concentration (~406 mg/kg DW) than their 50:50 (*v*/*v*) counterparts (~103 mg/kg DW). This shift confirms that more non-polar lipophilic structures (such as chlorophylls) exhibit enhanced solubility in higher organic solvent concentrations, whereas highly hydrophilic constituents favour a more aqueous 50:50 (*v*/*v*) environment. Nevertheless, when evaluated across all tested extraction protocols, the mechanical effects of UAE consistently produced the highest overall yields for several major classes. This trend is clearly illustrated in the late-season vegetative matrix (Herba October), where UAE optimized the recovery of total flavonoids to a peak of 169,798 mg/kg DW, outperforming conventional 50:50 (*v*/*v*) and 70:30 (*v*/*v*) hydroalcoholic maceration as well as continuous thermal reflux (Soxhlet) methods.

*Qualitative and Quantitative Evaluation of the Chromatographic Profile.* Comprehensive liquid chromatography–mass spectrometry (LC–MS) investigations conducted by [23] previously identified 43 distinct compounds within dandelion root and herba extracts, encompassing mono- and dicaffeoylquinic acids, tartaric acid derivatives, flavones, and flavonol glycosides, while establishing cichoric acid as the predominant constituent. These specific compounds are frequently documented as primary markers in dandelion extracts, with prior literature on leaves and roots emphasizing the prevalence of phenolic acids with high antioxidant activity, including both caffeic and chlorogenic acids [24]. Within the Radix and Mix samples, the confirmation of chlorogenic acid and caffeic acid represents a significant finding. The polyphenolic profile, determined by the RP-HPLC-DAD method for the analysed *T. officinale* samples, demonstrates partial alignment with established phytochemical literature. Historical data indicate that dandelion tissues typically exhibit a distinct profile of flavonoids—encompassing luteolin, luteolin-7-glucoside, luteolin-diglycosides, and chrysoeriol—alongside hydroxycinnamic acids, most notably cichoric, monocaffeoyltartaric, and chlorogenic acids [25]. In the present study, this chemical concordance is evidenced by the successful detection of luteol/luteolin within the Herba fraction and the confirmation of chlorogenic acid within both the Radix and Mix matrices. 

Recent quantitative and molecular dynamics assessments reinforce the therapeutic relevance of these molecules, demonstrating that chlorogenic, caffeic, and cichoric acids act as key active constituents responsible for the anti-inflammatory and radical-scavenging properties of the herb [55]. The quantitative results revealed a higher concentration of chlorogenic acid in the Mix than in the isolated Radix. This variation is scientifically plausible, as the mixed matrix incorporates aerial plant tissues (Herba) where literature consistently reports a superior total phenolic content compared to the subterranean organs.

Recent investigations continue to corroborate this complex distribution, also identifying an array of hydroxybenzoic and hydroxycinnamic acids, including gallic, syringic, vanillic, p-coumaric, caftaric, and chlorogenic acids across various plant tinctures and extracts [58]. Although cichoric acid could not be quantified in the current study due to the absence of a corresponding reference calibration curve, the appearance of intense chromatographic peaks within the 16.2–16.3 min retention time range—particularly prominent in the Flores and Mix fractions—strongly suggests the presence of major, unquantified hydroxycinnamic derivatives. This observation aligns with contemporary quantitative profiles characterizing cichoric acid as a primary diagnostic biomarker of *Taraxacum* species, which frequently exhibits prominent concentration peaks depending on regional and seasonal conditions [59].

Conversely, analysis of the Flores fraction indicated a profile rich in compounds absorbing at 274 and 280 nm wavelengths, characterized by elevated concentrations of gallic acid, kaempferol, and syringic acid. However, established literature regarding dandelion inflorescences focuses predominantly on complex flavonoids and luteolin derivatives rather than high concentrations of gallic acid and kaempferol [3]. The remarkably high values recorded for gallic acid and kaempferol must be interpreted with caution; while they may represent genuine quantitative variations, they may also be artificially inflated due to chromatographic coelution phenomena.

In characterization, the HPLC-DAD analysis of the *T. officinale* L. samples revealed a complex distribution of phenolic and flavonoid compounds, highlighting distinct organ-specific variations among the individual fractions:

Herba: gallic acid, kaempferol, luteol/luteolin, syringic acid, p-coumaric acid, and cinnamic acid were successfully resolved. Additionally, unassigned chromatographic peaks at 3.238 and 4.805 min potentially indicate the presence of unresolved caffeoylquinic or caffeic acid derivatives;

Radix: confirmed the presence of chlorogenic and caffeic acids, alongside gallic acid and kaempferol;

Flores: exhibited high concentrations of gallic acid, kaempferol, 3-O-methylgallic acid, syringic acid, and cinnamic acid, as well as a major unquantified peak at 16.270 min likely corresponding to a hydroxycinnamic derivative of the cichoric or dicaffeoyltartaric acid type;

Mix: Demonstrated the highest overall phytochemical complexity, successfully integrating constituents from multiple plant parts, including chlorogenic acid, caffeic acid, *p*-coumaric acid, tentative caftaric acid, 3-*O*-methylgallic acid, kaempferol, and putative luteolin derivatives.

Ultimately, these experimental findings are partially consistent with classical and updated literature, which designates cichoric acid, monocaffeoyltartaric acid, chlorogenic acid, caffeic acid, and luteolin derivatives as the primary chemical markers of the species [3,23,24,25,55,59]. Taken together, these results provide an empirical guide to the optimal extraction conditions and harvesting strategies required to selectively target each bioactive compound from the *Taraxacum* plant matrix.

The obtained results indicate these guides (Table 14) to the optimal conditions for extracting each target compound from the *Taraxacum* plant.

This study provides strong empirical validation for two primary pharmacognostic recommendations: the traditional utilization of whole-plant *Taraxacum* extracts to ensure bioactive stability and synergy, and the strategic integration of an autumn harvest with UAE to achieve the optimal pharmacological valorization of this botanical resource.

### 3.4. Evaluation of the Antimicrobial Effect of Herbal Extracts

The observed distribution of microbial sensitivity across the analysed *T. officinale* L. extracts is highly consistent with fundamental microbiological principles. Gram-positive bacteria (*S. aureus* and *E. faecalis*) lack a protective outer lipid membrane, leaving their peptidoglycan-rich cell walls structurally vulnerable. This structural vulnerability facilitates the penetration and subsequent cellular cross-linking induced by the high concentrations of tannins and flavonoids found within the Herba and Mix, October and May samples. The strong bactericidal and bacteriostatic effects observed against *S. aureus* and *E. faecalis* are well-supported by existing literature [59,60], which previously demonstrated that the intricate polyphenolic content of *T. officinale* L. aerial parts exhibits potent inhibitory activity against *S. aureus*. Furthermore, the consistency between the MIC and MMC values for *E. faecalis* across several extracts (including Herba 50% and Flores UAE) strongly underscores a robust, targeted bactericidal mechanism against this specific pathogen. 

The in vitro antimicrobial assessment of the May extracts reveals a unique pharmacological profile compared to the autumnal harvest. This selective antibacterial profile, coupled with an absolute lack of antimycotic efficacy against *C. albicans*, is strongly associated with the seasonal peak of hydrolysable tannins observed during the phytochemical profiling of young, early-season plant tissues. The rigid chitin-glucan structure of the fungal cell wall remains highly resistant to the specific phenolic compounds isolated utilizing standard ethanol-water mixtures [60], thereby confirming the specialized antibacterial selectivity of these preparations. The comprehensive profiling of *T. officinale* L. extracts reveals a direct correlation between the quantified phytochemical matrix and the observed in vitro antimicrobial behaviours.

In contrast, the heightened resistance demonstrated by the Gram-negative strains (*P. aeruginosa* and *E. coli*) is biologically plausible due to their complex outer lipopolysaccharide (LPS) envelope, which acts as a formidable permeability barrier to many hydrophilic plant metabolites [61,62,63,64]. Literature indicates that plant extracts mainly act as bacteriostatic agents by inhibiting enzymes [62], affecting membranes sub lethally, chelating metals [63], or inducing oxidative stress [64]. Complete bactericidal activity occurs less frequently and typically requires higher concentration, extract fractionation/standardization, or further processing. Furthermore, *P. aeruginosa* cells possess highly efficient efflux systems and intrinsic multi-drug resistance mechanisms that actively restrict the intracellular accumulation of exogenous agents [65,66,67,68]. This explains why the 50:50 (*v*/*v*) ethanol extracts, while effective against Gram-positives, generally fell short of overcoming the structural barriers of most Gram-negative strains, resulting in narrower zones of inhibition for the 50:50 (*v*/*v*) floral fractions. However, the unique, targeted efficacy of the Flores UAE extract against these resistant Gram-negative strains highlights the distinct pharmacological value of the floral organs. This phenomenon aligns with prior investigations documenting that highly concentrated, specific lipophilic flavonoid fractions—such as luteolin and its derivatives, which uniquely peak in dandelion inflorescences—possess the necessary partition coefficients to interact with, physically permeabilize the outer LPS layer, and disrupt Gram-negative multidrug efflux pumps [69,70,71]. Characterization of the MICMA parameter provides crucial insights into the anti-biofilm dynamics of these Phyto preparations. When the MICMA falls below the standard MIC, it strongly indicates that the extracts interfere with initial bacterial attachment mechanisms and the early stages of biofilm development, rather than exerting direct bactericidal action. While this anti-adherence profile was largely narrow and restricted to Gram-positive strains due to the moderate baseline concentration of active phenolics, the ability of the Flores UAE extract to deter Gram-negative cell adherence (e.g., *E. coli*) suggests that ultrasonic extraction successfully releases specialized compounds capable of disrupting early attachment phases even in highly guarded outer membranes.

The distinct biological fingerprints of the extracts are intrinsically linked to their ontogenetic stage, anatomical origin, and the specific thermodynamic parameters of their extraction, establishing clear structure-activity relationships (SAR) that dictate their pharmacological efficacy.

A positive correlation exists between tannin concentration and the inhibition of *E. faecalis*, driven by the capacity of tannins to interact with and disrupt bacterial cell wall proteins [72,73,74,75]. The Radix UAE extracts, characterized by the highest recorded tannin levels, achieved the most potent MIC at 125 µL/mL. This relationship is supported by [76] Borges, who noted that phenolic acids and tannins, such as gallic acid, act by precipitating membrane proteins and increasing the permeability of the bacterial cell wall. In the *Taraxacum* root, these complex polyphenols drive the disruption of the robust cell wall characteristic of *Enterococcus* species. Furthermore, extracts with elevated anthocyanin levels (e.g., Radix UAE) showed the lowest microbial adherence values at 62.5 µL/mL for *S. aureus*. While anthocyanins may not serve as primary bactericidal agents, they play a critical role in anti-virulence by interfering with the initial adherence of pathogens to surfaces. By preventing this initial phase, the extract inhibits subsequent biofilm formation, a critical factor in treating persistent infections.

The antimicrobial activity of *Taraxaci herba* is primarily directed toward Gram-positive pathogens, with *S. aureus* demonstrating the highest sensitivity. Quantitative analysis revealed a linear correlation between flavonoid concentration and IZD. The Herba May UAE extract, exhibiting the highest flavonoid recovery, produced the largest IZD (9.66 ± 0.57 mm) and a matching MIC of 125 µL/mL. This aligns with benchmarks established by [77] Diaz regarding the inhibitory potential of dandelion extracts, confirming that foliar flavonoids serve as primary killing agents by disrupting cytoplasmic membrane function. However, Herba extracts showed limited efficacy against *E. coli* and *P. aeruginosa*. This selective resistance is attributed to the relative scarcity of high-molecular-weight tannins in the leaves compared to the roots, rendering the leaf extracts less capable of penetrating the complex outer LPS membrane of Gram-negative bacteria. The Mix extract exerts simultaneous pressure on multiple bacterial targets, reflecting a “sum-of-parts” synergy where flavonoids disrupt enzymatic activity while tannins compromise membrane integrity [77]. This multi-target approach resulted in consistent MIC values of 125 µL/mL against *E. faecalis* and robust anti-adherence properties, serving as an effective agent for preventing the transition of pathogens from planktonic growth to sessile, antibiotic-resistant biofilm states.

The efficacy of *Taraxaci* inflorescences results from a unique synergy between polyphenols and high carotenoid levels, which peaked at 93.71 mg/kg in the May Soxhlet extract. While flavonoids disrupt the cytoplasmic membrane, high concentrations of carotenoids further destabilize the bacterial lipid bilayer, facilitating the entry of polar bioactive components into the cell. This effect was particularly evident against *E. faecalis*, where MIC values improved fourfold in direct correlation with increased carotenoid yields. The synergistic antimicrobial efficacy of the extract relies on distinct, complementary interactions with the bacterial cytoplasmic membrane. Flavonoids generally possess a polar structure due to their numerous hydroxyl (−OH) groups, allowing them to target the hydrophilic (water-attracting) phosphate heads on both the outer and inner surfaces of the membrane bilayer [78]. Through hydrogen bonding and electrostatic interactions, flavonoids bind to these polar head groups, perturbing the membrane’s surface tension and inducing localized packing defects [79]. This initial surface disruption alters overall membrane fluidity, induces the leakage of small intracellular ions (such as $K^{+}$), and disrupts the membrane potential (Δψ), ultimately weakening the structural integrity of the cellular boundary.

Concurrently, carotenoids—such as β-carotene and lutein—act as highly lipophilic, long-chain hydrocarbon molecules that target the hydrophobic (water-repelling) core of the lipid bilayer where the fatty acid tails reside [80,81]. Due to their hydrophobic nature, carotenoids easily partition deep into the center of the membrane matrix. At high concentrations, these elongated molecules intercalate between the fatty acid tails, forcing the tightly packed lipids apart [82]. This localized expansion severely fluidizes and destabilizes the core structure, essentially “melting” or thinning the membrane. Consequently, the rigid lipid barrier loses its foundational function, creating transient pores and structural gaps that accelerate cell death.

The biological data remains tightly consistent with the phytochemical variations determined by the extraction conditions, highlighting the critical role of processing parameters. The enhanced antimicrobial efficacy observed with the 70:30 (*v*/*v*) ethanolic extracts compared to the 50:50 (*v*/*v*) formulations underscores the significant influence of solvent polarity on the isolation of bioactive compounds. Phytochemical extraction mechanics demonstrated that 70:30 (*v*/*v*) ethanol was significantly more efficacious at isolating moderately non-polar and hydrophobic molecules compared to the highly aqueous 50:50 (*v*/*v*) solution. Because the specific antimicrobial agents driving bioactivity—namely, complex tannins and lipophilic flavonoids—require a comparatively lower-polarity solvent for optimal dissociation from the plant matrix, the 70:30 (*v*/*v*) hydroalcoholic solvent successfully sequestered these critical agents, translating directly into enhanced in vitro efficacy [83,84]. Furthermore, UAE consistently exhibited broader-spectrum antimicrobial activity and larger inhibition zones compared to its Soxhlet counterparts. The quantitative phytochemical data demonstrated that prolonged thermal stress during Soxhlet extraction induced massive degradation of sensitive molecules, evidenced by the severe pheophytinization of chlorophyll and the precipitous oxidative destruction of ascorbic acid [76,85]. This thermodynamic degradation extends to thermolabile polyphenolic antimicrobial agents. Thus, the diminished bioactivity of the Soxhlet samples mirrors their compromised, heat-denatured phytochemical profile, whereas the superior biological efficacy of the UAE samples reflects their intact, holistically preserved bioactive matrix [86,87]. Interestingly, the sustained activity of certain Soxhlet extracts (such as an IZD of 8.33 ± 0.57 mm 8.33 against *S. aureus* suggests that specific dandelion flavonoids possess significant inherent thermal stability, retaining their ability to inhibit nucleic acid synthesis and disrupt cytoplasmic membranes even under rigorous, continuous extraction conditions [88]. The lack of distinct variance between the isolated Herba and the blended Mix parameters suggests that the incorporation of diverse plant tissues in the Mix matrix achieves a comparable threshold of active compound concentration and diversity without introducing antagonistic chemical interferences. Taken together, these findings establish clear optimization guides for matching harvest timelines and processing technologies to specific therapeutic targets. The polarity-driven selectivity is explicitly evidenced by the distinct bioactivity variations observed between the hydroalcoholic cohorts. While ethanol functions as an eco-friendly ‘green’ solvent capable of isolating a broad spectrum of metabolites [89], matching the exact water-to-organic ratio to analyte polarity remains critical. The highly polar 50:50 (*v*/*v*) ethanol system preferentially isolates hydrophilic matrix elements such as polysaccharides and highly polar organic acids, which frequently lack primary antimicrobial properties. Conversely, the 70:30 (*v*/*v*) system facilitates the dissolution of intermediate-polarity secondary metabolites, including aglycone flavonoids, triterpenes, and specific sesquiterpene lactones [90].

### 3.5. Evaluation of the Cytotoxic Potential by Brine Shrimp Lethality Assay (BSLA)

The Brine Shrimp Lethality Assay (BSLA) serves as a fundamental diagnostic tool for preliminary screening of plant extracts to determine their cytotoxic potential. The selection of larvae at the 24 h post-hatching stage is strategically dictated by their metabolic profile; at this developmental juncture, the organisms possess endogenous energy reserves sufficient to sustain essential ontogenetic processes.

This nutritional autonomy provides a controlled experimental framework where the biological response and induced ethological modifications can be isolated with high precision. By utilizing this specific window, the assay ensures that the observed effects are not confounded by external nutritional factors or chemical variables in the test medium. Consequently, any detected cytotoxic behaviour can be confidently attributed to the direct interaction between the vegetal extract and the larval biological system.

The integrated assessment of *T. officinale* L. extracts demonstrates that while all vegetal organs possess bioactive properties, their impact on larval development is highly specific. Although all extracts were classified as non-toxic according to Clarkson’s criterion—with LC_50_ values significantly exceeding the 1000 µg/mL threshold—they induced measurable physiological stress manifested as growth inhibition [91].

Radix extract emerged as the most potent inhibitor, resulting in a Relative Growth in Length (RGL) deficit of −350.45 µm compared to the control group. This finding is corroborated by the daily growth kinetics, where Radix-treated larvae (LR1, LR10) exhibited a minimal growth rate of approximately 1, contrasting with 11–16% growth rates observed in aerial and mixed extracts. The heightened potency of the root extracts is likely attributable to high concentrations of specialized bitter principles, specifically sesquiterpene lactones such as taraxacin and taraxacoside. These compounds are documented by [92] for their cytotoxic properties, which potentially induced the arrested development observed in nauplii. These findings align with the literature [93,94], which documented the inhibitory effect signalling pathway, a critical mediator of cellular differentiation. The markedly stunted growth observed in larvae exposed to root-derived extracts is consistent with these reported biochemical properties of taraxasterol [95,96]. Furthermore, the presence of inulin and related complex carbohydrates may have modulated the physicochemical properties of the test medium, such as osmotic balance and viscosity, thereby impeding the larval moulting process or increasing metabolic expenditure. Notably, the known hypoglycaemic activity [97,98] of extracts and also caffeic acid might provide a physiological basis for the observed larval tremors, suggesting a disruption in systemic energy homeostasis.

The functional equivalence (*p* > 0.05) observed between Herba, Flores, and Mix extracts suggests a redundant phytochemical profile in aerial tissue rich in tannins and ascorbic acid, which exerts a less severe developmental arrest. Ultimately, the test highlights that the subterranean organs of *T. officinale* L. harbour a unique phytochemical synergy that significantly disrupts larval ontogeny without reaching acute lethal thresholds.

These results indicate non-toxic effects after Clarkson’s toxicity criterion, and it is an important detail that these extracts can be used as natural alternatives for therapies. Such a favourable safety profile reinforces the viability of the studied botanical species for integration into phytotherapeutic applications, as the concentration required to induce significant biological stress far exceeds typical therapeutic dosages.

## 4. Materials and Methods

Mature specimens of *T. officinale* L. were collected during two periods, April–May and September–October 2024, from the Moldavian Central Plateau (47°02′57.6″ N 26°44′43.7″ E), located in the northeastern region of Neamt County, Romania (broadly bounded by 48°15′ N 26°43′ E to the North, 43°40′ N 25°22′ E to the South, 46°08′ N 20°19′ E to the West, and 45°09′ N 29°40′ E to the East). Geologically, the region consists of sand and gravel strata overlain by loessoid clay deposits, and it is characterized by a moderate temperate continental climate. The mean annual temperature ranges from 8 °C to 9 °C, with average temperatures of 14–16 °C in May and 12–14 °C in October. The mean annual precipitation is approximately 550–600 mm, recording values of 20–30 mm/24 h during the collection months. The harvested biological material, collected at full maturity, was categorized into four distinct groups: roots (coded as Radix), aerial parts comprising stem and leaves (coded as Herba), inflorescences (Flores), and whole plant (coded as Mix). Following separation, the anatomical parts were thoroughly washed with tap water, dried at ambient temperature on metal sieves, and subsequently ground into a fine powder.

### 4.1. Experimental Procedures for Phytochemicals Extraction

To evaluate the efficiency of bioactive compound recovery from *Taraxacum* plants, three distinct extraction methodologies were employed: conventional maceration, Soxhlet extraction, and non-conventional method ultrasound-assisted extraction (UAE).

#### 4.1.1. Conventional Cold Maceration

At ambient temperature, vegetal fluid extracts were prepared via traditional cold maceration to serve as a baseline for comparison. Dehydrated *Taraxacum* specimens were processed into fine powders and categorized by organs: Radix (roots), Herba (stem and leaves), Flores (inflorescences), and Mix (aerial parts and roots). Samples (10 g) were suspended in 100 mL of hydroalcoholic solvents at two concentrations: 50:50 (*v*/*v*) and 70:30 (*v*/*v*) ethanol. The extraction was conducted in amber conical flasks to prevent photodegradation. The mixtures were homogenized manually and maintained at ambient temperature for 14 days with intermittent agitation. Following the maceration period, the extracts were clarified via gravity filtration using Whatman quantitative filter paper (Cytiva, Little Chalfont, Buckinghamshire, United Kingdom). The resulting filtrates exhibited a chromatic range from light brown to olive-green, correlating with the specific plant organ and solvent polarity.

#### 4.1.2. Soxhlet Extraction

For a comparative analysis of exhaustive extraction, a Soxhlet apparatus (Electrothermal, Stone, Staffordshire, United Kingdom) was utilized. A quantity of 10 g of Taraxacum powder from each category was loaded into a cellulose thimble and placed inside the extraction chamber. An aqueous ethanol solution (70:30 *v*/*v*) was employed as the extraction solvent. The process was maintained under continuous reflux for 4 h, ensuring repeated cycles of solvent contact with the solid matrix. Post-extraction, the solution was filtered through Whatman blue band filter paper, yielding an ochre-brown extract.

#### 4.1.3. Ultrasound-Assisted Extraction

UAE was implemented as a high-efficiency, “green” alternative to conventional methods. Aliquots of 10 g from each plant category (Radix, Herba, Flores, and Mix) were submerged in 100 mL of 70:30 ethanol (*v*/*v*) within a 200 mL borosilicate flask. The samples were processed using a Hielscher Ultrasonic Processor UP200Ht, Germany (200 W, 26 kHz), for 30 min at a controlled temperature of 25 °C. This technique leverages the principle of acoustic cavitation to facilitate cell wall disruption and enhance the mass transfer of intracellular bioactive principles into the solvent.

### 4.2. Experimental Procedures for Hydroalcoholic Extracts of T. officinale Evaluation

#### 4.2.1. Physicochemical Evaluation of Hydroalcoholic Extracts

The physicochemical profile of the hydroalcoholic extracts was assessed by determining the pH, electrical conductivity (EC), total soluble solids (Brix degrees), and relative density. All electrochemical measurements (pH and EC) were performed using a digital multiparameter Hanna Instruments Edge Benchtop multiparameter (Hanna Instruments, Woonsocket, RI, USA).

The pH values were determined via the direct potentiometric method using a combined glass electrode equipped with an automatic temperature compensation (ATC) system. Before analysis, the instrument was calibrated using a two-point calibration protocol with standard buffer solutions (pH 7.01 and 10.01). All measurements were conducted at room temperature (20 ± 1 °C) after achieving complete stabilization of the potentiometric signal, exhibiting an electrode slope of 94.5% and an offset of −3.0 mV.

The electrical conductivity of the hydroalcoholic extracts was measured using a direct conductometric method. The multiparameter meter was fitted with a shielded four-platinum-ring conductivity probe (EC probe) featuring an internal temperature sensor for automatic temperature compensation (ATC). Calibration was performed prior to testing using a certified reference solution with a known conductivity of 1413 µS/cm (at 25 °C). The assessments were carried out at (20 ± 1 °C), and the final stable galvanic values were recorded in µS/cm.

The total soluble solids content, expressed in degrees Brix (°Brix), was evaluated using a portable analogue optical refractometer Kern ORA 32BA (Kern & Sohn GmbH, Balingen, Germany). The device was equipped with an ATC system and featured a measurement scale of 0–32 °Brix. A drop of each hydroalcoholic extract was applied onto the main prism, and the boundary line was directly read from the graduated scale.

The relative density of the plant extracts was determined by the standard pycnometric method, using a glass pycnometer equipped with a ground capillary stopper, which was calibrated beforehand with high-purity distilled water at the reference temperature. The experimental procedure involved the successive gravimetric determination of the empty pycnometer, the pycnometer filled with the reference aqueous phase, and the pycnometer filled with the respective *T. officinale* extracts. All weighings were performed on a high-precision analytical balance. The relative density, expressed in g/cm^3^, was calculated as the ratio between the mass of the extract volume and the mass of an equivalent volume of distilled water at the working temperature (20 ± 1 °C).

#### 4.2.2. Mineral and Heavy Metals Content Evaluation of Hydroalcoholic Extracts

Before analysis, the collected plant organs, pre-cut into small fragments, were oven-dried at 80 °C for 72 h until a constant weight was achieved. An aliquot of 0.25 g from each dried sample was subjected to wet acid digestion using 5 mL of concentrated nitric acid (HNO_3_). The mixture was heated and boiled at 150 °C for 1 h. After cooling to room temperature, 2 mL of hydrogen peroxide (H_2_O_2_, 30%) was added to ensure complete mineralization of the organic matrix, followed by an additional boiling step at 150 °C for 2 h [99,100]. To minimize chemical matrix interferences during spectrometry, the digested solutions were quantitatively transferred and diluted to a final volume of 50 mL using a 2% NH_4_Cl solution and a 0.5% CaCl_2_ solution, respectively [101,102,103,104]. The concentrations of target metals in the digested matrices were quantified via Flame Atomic Absorption Spectrometry (FAAS) using a High-Resolution Continuum Source AAS Spectrometer (HR-CS AAS, ContrAA 700, Analytik Jena AG, Germany). An air-acetylene flame technique was employed at specific resonance wavelengths optimized for each metal: Cu 324 nm, Mn 279 nm, Zn 213 nm, Fe 248 nm, Cd 228 nm, Pb 283 nm, Ni 232 nm [105,106,107]. Quantitative determination was performed against external multi-point calibration curves plotted for each selected metal (Cu, Mn, Zn, Fe, Cd, Pb, Ni), as illustrated in Figure S1. All final elemental concentrations were processed and expressed in mg/kg D.W.

#### 4.2.3. Experimental Procedures for Determination of Extracts Bioactive Compounds

For total phenolic compounds determination, a UV-Vis spectrophotometric version of the Folin-Ciocâlteu method was used: 1 mL vegetal extract was reacted with 5 mL Folin-Ciocâlteu reagent (10%) and 4 mL sodium bicarbonate solution (7.5%) for 30 min. Spectrophotometric absorbance was measured at 765 nm wavelength against a blank. A calibration curve was prepared by using different gallic acid concentrations [108,109,110]. Concentrations were expressed as mg/kg dry weight gallic acid equivalent (GAE) for total phenolic compounds.

For determination of chlorophylls and total carotenoid concentration, 1 mL plant extract was diluted in 9 mL 80% acetone (triplicate samples for each species). The resulting extract was filtered under normal pressure through Whatman blue-banded filter paper, and the spectrophotometric absorbance was read (using a UV-Vis S106 WPA spectrophotometer, Biochrome Ltd., Cambridge, UK) against an 80% acetone blank at 470 nm, 647 nm, and 663 nm wavelengths [111,112]. The absorbance values were used to calculate chlorophyll a/chlorophyll b/carotenoid concentration, according to the specific trichromatic equations [113,114].

For determining the flavonoid content, 1 mL of the extract was diluted in 4 mL of methanol and filtered (triplicate samples). Then, 0.5 mL of extract was diluted in 4 mL of water, and an 8 mL methanol mixture, and the spectrophotometric absorbance was read against a methanol: water blank at 340 nm wavelength [115,116,117,118].

For determining the concentration of tannins 0.5 mL of vegetal extract was diluted in 2 mL of distilled water. Next, 1.25 mL of 25% gelatine and 2.5 mL saturated solution acidified with 1% NaCl were added, followed by 30 min stirring and filtration, Folin-Ciocâlteu. Spectrophotometric absorbance was read at 765 nm wavelength [119]. The value was subtracted from the concentration of total phenolic compounds, determined by reacting 1 mL of each plant extract with 5 mL Folin-Ciocâlteu reagent (10%) and 4 mL sodium bicarbonate (7.5%), and reading the absorbance at 765 nm wavelength, compared to a calibration curve made with gallic acid [120].

For determining the concentration of anthocyanins: absorbance of the extract was read at 520 and 700 nm wavelengths [121].

For total ascorbic acid, 1 mL of ethanol extraction, reaction with 7.4 mL ammonium molybdate 5%, and 1.6 mL sulfuric acid 5%, 30 min incubation followed by spectrophotometric reading at 494 nm wavelength was employed [122].

#### 4.2.4. Chromatographic Separation, Characterization, and Quantitative Fingerprinting of Polyphenols in *Taraxacum officinale* UAE Extracts via Validated RP-HPLC-DAD

In this research, a reversed-phase high-performance chromatography (RP-HPLC) method with diode array detection (DAD) was optimized and adapted, starting from the standardized methodology described in the USP monograph 30-NF25 [123] for the determination of total polyphenols. Chromatographic analyses were carried out on an Agilent 1200 HPLC instrumentation suite comprising a quaternary gradient delivery pump, an inline micro-vacuum degassing unit, an automated sample injection system (autosampler), a column thermal stabilization compartment, and a high-resolution diode array detector (DAD). Solute separation was performed using a standard C18 stationary phase column (150 mm × 4.6 mm, 5 µm particle size; Zorbax XDB or validated functional analogue) strictly stabilized at an operational temperature of 35 °C. Mobile-phase elution was performed in gradient mode with a binary system containing Solution A (0.1% aqueous phosphoric acid, *v*/*v*) and Solution B (HPLC-grade pure acetonitrile). Operating parameters included a steady flow rate of 1.5 mL/min and a fixed sample injection volume of 20 µL. Spectral acquisition focused on a central channel of 310 nm wavelength to accommodate the major target phenolic chromophores, finishing the total analytical screening window in 22 min. The gradient timeframe is provided in Table 15.

To isolate and refine the analyte fraction before instrument entry, a 5 mL aliquot of the raw liquid extract was filtered through a 0.45 µm pore size Millipore membrane filter, followed by direct autosampler injection. Concurrently, a composite reference solution encompassing all 23 pure phenolic target standards was prepared to evaluate baseline retention and validate quantitative performance. System precision and peak placement reproducibility were examined through 6 consecutive replicate injections. Data were subjected to Analysis of Variance (ANOVA), Welch’s *t*-test, and Tukey HSD with *p* < 0.05 to identify significant differences between experimental cohorts. A *p*-value of < 0.05 was established as the threshold for statistical significance. The analysis was performed with Python software (version 3.10, Python Software Foundation, Beaverton, OR, USA).

### 4.3. Qualitative Evaluation of Antimicrobial Activity

For qualitative testing of antimicrobial activity, microbial suspensions were prepared and adjusted to 1.5 × 10^8^ CFU/mL according to the 0.5 McFarland nephelometric standard from 18 to 24 h cultures developed on solid media (Muller–Hilton for bacteria and Sabouraud for yeast). The strains were obtained from the Microbial Strain Collection of Faculty of Biology, University of Bucharest, Romania, and confirmed by MALDI-TOF.

Antimicrobial activity was determined by the adapted diffusion method, standardized for the control of the antimicrobial activity of antibiotics, by CLSI (Clinical and Laboratory Standards Institute). A specific solvent control for the extracts was used for all variants. Then, 10 μL of each extract was spotted on the medium seeded on the cloth. The appearance of an inhibition zone at the level of the sample spot placed on the culture medium was considered a positive result, and the diameters were measured.

#### 4.3.1. Qualitative Evaluation of Antimicrobial Activity (Minimum Inhibitory Concentration—MIC)

Antimicrobial activity was quantitatively assessed using the minimum inhibitory concentration (MIC). The minimum inhibitory concentration is the lowest concentration of extract that can stop the growth of microorganisms and was determined quantitatively on 96-well plates using the serial microdilution method in liquid medium. Each extract was serially diluted in a binary way in a volume of 100 μL of medium. Subsequently, the wells were inoculated with 10 μL of microbial suspension with a density of 1.5 × 10^8^ CFU/mL (CFU—colony-forming units). The microbial suspensions were prepared in sterile physiological saline from 24 h cultures. Plates were incubated at 37 °C for 24 h, and data were examined using macroscopic examination and 620 nm wavelength absorbance reading. Negative (uninoculated media, sterility control), positive (inoculated media without treatment), and solvent controls were used. The analysis was performed in duplicates.

#### 4.3.2. Evaluation of the Minimum Microbicidal Concentration

To determine the minimum microbicidal concentration (MMC), a 5 μL aliquot was sampled from each microtiter well showing no visible growth—as determined by absorbance readings at 620 nm using a Flex Station 3 UV-Vis spectrophotometer (Molecular Devices, San Jose, CA, USA)—and spotted onto solid media (Mueller–Hilton agar for bacterial strains and Sabouraud dextrose agar for yeast strains). The plates were incubated for 24 h at 37 °C. The MMC was defined as the lowest concentration at which no microbial colonies were visually observed on the solid surface. 

#### 4.3.3. Evaluation of the Capacity to Inhibit Microbial Adherence

Microbial cells were cultured in 96-well plates with nutrient broth in the presence of the tested compounds. Following the MIC readings, the plates were emptied and washed three times with sterile physiological saline. Adherent cells were fixed with cold methanol for five minutes, followed by staining with a 0.1% crystal violet solution for fifteen minutes. The staining solution was then removed, and the plates were washed three times. Microbial biofilms formed on well plates were resuspended in 33% acetic acid, and the intensity of the coloured suspension was evaluated by reading the absorbance at 492 nm wavelength, using the same Flex Station 3 UV-Vis spectrophotometer.

Data were subjected to Analysis of Variance (ANOVA), Welch *t*-test, Tukey B, Mann–Whitney and Kruskal–Wallis, with *p* < 0.05 to identify significant differences between experimental cohorts. A *p*-value of < 0.05 was established as the threshold for statistical significance. The analysis was performed with Python software (version 3.10, Python Software Foundation, USA).

### 4.4. In Vivo Brine Shrimp Lethality Assay (BSLA)

The cytotoxicity BSLA test was performed according to the Artoxkit protocol, ARTOXKIT M: 24 h mortality test based on the *Anostraca* crustacean *Artemia salina* (Figure S7). This assay adheres to ASTM Standard Guide E1440-91 [124]. 

#### 4.4.1. Preparation of Test Solutions

Alcoholic solutions of plant extracts-Radix, Herba, Flores, Mix-were diluted in 10 ppt saline water in a ratio of 1:10. From the stock solutions, volumes of 10, 20, 30, 50, 80, 100, 400, 500 µL/mL (1, 2, 3, 5, 8, 10, 40, 50 mg/mL) were tested. The testing was performed in stages until quantifiable effects were obtained.

Hatching and preparation of larvae for testing *Artemia* cysts were incubated in artificial saline water (30–32 ppt), at a temperature of 22–23 °C, for 48 h, under conditions of continuous aeration and lighting. The 24 h larvae (nauplii) were separated using a binocular magnifying glass and introduced into the test tanks.

#### 4.4.2. Measurement of Induced Acute Mortality (Cytotoxicity)

The testing was conducted using microplates, and Plexiglas wells with volumes of 1 mL were used. Larvae at the nauplius stage were isolated and transferred into 12-well test chambers. Each replicate contained a population density of 10–20 nauplii per well. To initiate the exposure, test solutions were introduced to the chambers, maintaining a final volume of 1 mL. The negative control was represented by water solutions. Tests were also performed for ethanol controls (10, 20, 40, 50 µL/mL), the 70:30 ethanol solution being introduced into the tanks with larvae and saline water. The larvae were not fed, and the experiments were carried out at a constant temperature of approximately 22 ± 1 °C. The evaluation of the effects was carried out at different intervals of 24 h, 48 h, 72 h, and 96 h. During the tests, the following variables were monitored: larval behaviour (antenna movements, feeding, moulting), length growth (µm), and the rate of larval growth changes compared to the larvae in the control samples, mortality/viability. Lethal effects were quantified based on observations made with binoculars (×4). Larvae that remain on the bottom of the vessel and no longer show specific movements (appendage movement) will be considered dead. The evaluation was carried out according to the formula:

Mortality (M) of larvae (%) = (number of dead larvae from samples − number of dead larvae in control) × 100/total number of larvae; Viability (%) = 100 − M

#### 4.4.3. Measurement of Larval Growth

Larval growth was assessed by determining total body length (µm) from a representative sample of n = 20 specimens per experimental group. Following the exposure period, larvae underwent preparation for microscopic examination and were imaged with specialized digital imaging software calibrated (OPTIKA ProView digital camera software (version 3.7, OPTIKA S.r.l., Ponteranica, Italy)) for each magnification, ensuring spatial measurement accuracy.

Relative Growth in Length (RGL) was calculated to determine the percentage of growth inhibited or stimulated relative to the control group over the 4-day exposure period.

The RGL is defined by the following equation:

$$\mathrm{RGL}\;(\%)\;=\;\frac{LP-LC4}{LC4-LC1}\;\times \;100$$

where

LP: Mean length (µm) of the larvae in the experimental samples (4 days of exposure, first day was ignored), LC4: Mean length (µm) of larvae in the control group after 4 days, LC1: Initial mean length (µm) of larvae at the start of the experiment (Day 1).

To evaluate the daily growth velocity, Daily Relative Growth Rate (RGLD) was derived:

$$\mathrm{RGLD}\;(\%)\;=\;\frac{RGL}{4}$$

Data were subjected to a one-way Analysis of Variance (ANOVA) to identify significant differences between experimental cohorts. A *p*-value of <0.05 was established as the threshold for statistical significance.

Dose–response modelling: the probit regression model was employed to determine the median lethal concentration (LC_50_), representing the concentration required to induce 50% mortality in the test organisms.

Principal component analysis (PCA): to elucidate the complex interactions between the phytochemical profile of the extracts and the observed biological responses, PCA was performed. This multivariate approach integrated the following variables: toxicological potency (LC_50_), growth kinetics (RGLD), and larval viability across specific concentration gradients (VL10, VL40, VL50).

This analysis was performed with Stat Plus: Mac software (Stat Plus: Mac, Analyst Soft Inc.), a statistical analysis program for macOS (version v8; Analyst Soft Inc., Brandon, FL, USA).

## 5. Conclusions

The comprehensive profiling of *Taraxacum officinale* L. across its various anatomical organs and ontogenetic stages reveals a sophisticated biochemical strategy that dictates its pharmacological potential. This study establishes that the therapeutic value of dandelion is not a static attribute, but a dynamic variable shaped by the interplay of plant organ, harvest season, and extraction methodology. Findings indicate that therapeutic value is maximized through targeted selection: spring inflorescences and leaves serve as primary reservoirs of flavonoids and Vitamin C, while autumn roots concentrate condensed tannins and polysaccharides, which are essential for antimicrobial and astringent applications. Notably, the multi-component profile of whole-plant extracts may offer a protective microenvironment that helps stabilize thermolabile molecules like Vitamin C, pointing toward potential biochemical synergy. Although the precise role of unanalysed matrix components—such as lipids or pectins—in this stabilization remains to be fully investigated, these preliminary observations suggest that the total plant matrix warrants deeper chemical interaction studies.

Methodologically, UAE using 70:30 (*v*/*v*) ethanol was identified as the superior method, as it effectively shatters the plant matrix via acoustic cavitation without the thermal degradation associated with Soxhlet extraction. This approach preserves intermediate-polarity compounds as luteolin derivatives, which allows flower extracts to permeabilize the membranes of resistant Gram-negative bacteria, whereas autumn root extracts act mainly on Gram-positive pathogens. Furthermore, the presence of anthocyanins across all organs provides a significant anti-virulence effect by inhibiting biofilm formation. Toxicological analysis confirms the plant’s safety, showing a total absence of heavy metals and non-toxic LC_50_ values (exceeding 1000 µg/mL). However, root extracts exhibit a unique biological fingerprint that induces measurable physiological stress in larval models, suggesting potential for targeted metabolic therapies.

This research provides a robust analytical framework for the standardization of dandelion-based products in the evolving field of evidence-based phytotherapy. From an analytical chemistry perspective, the most significant finding is the organ-specific chromatographic behaviour of chlorogenic acid and its derivatives. From a quality control and standardization perspective, the study highlights a critical optimization conflict between extraction efficiency and chromatographic resolution: whereas isolated floral profiles suffer from analytical overestimation due to coelution, combining organs into a Mix matrix successfully balances out individual tissue anomalies, offering the most stable and representative chemical fingerprint for legal monograph standardization.

The study concludes that a general extraction protocol is inefficient for recovering the full phytochemical spectrum of *T. officinale* L. Instead, a dual-harvest strategy is proposed for optimal pharmacological valorisation of *T. officinale* L. It recommends a strategic integration of either an autumn harvest paired with UAE for defence-oriented extracts, or a spring-harvested whole plant to maximize antioxidant synergy. By selecting the flower-specific UAE extract for Gram-negative challenges or the whole-plant Mix for synergistic biofilm prevention, clinicians and researchers can use standardized dandelion extracts as a robust, safe, and natural alternative to traditional antimicrobial therapies. In conclusion, *Taraxacum officinale* L. is not merely a common weed, but a valuable therapeutic biofactory.

## Figures and Tables

**Table 1:** Influence of regional environmental characteristics on heavy metal concentrations across various international sites.

Element	Romania (Năvodari, 2013) [14]	Poland (Warsaw/ Urban, 2000) [15]	Italy (Mining Sites 2012) [16]	SUA (EMA, 2009) [13]	Bulgaria (Mining Sites, 2023) [19]
Copper (Cu)	9.08–10.27	13.0	Up to 64	2–58	High
Zinc (Zn)	22.81–24.60	67.0	Up to 189	18–261	High
Lead (Pb)	4.08–9.28	7.6	Up to 193	0.5–45	High
Cadmium (Cd)	0.008–0.031	0.97	N/A	0.55–3.11	>5.0 (Alarming)
Nickel (Ni)	0.33–0.35	1.9	N/A	N/A	0.33–0.35
Iron (Fe)	N/A	310.0	N/A	N/A	N/A
Characteristic	industrial area, alkaline soils, moderate accumulation	traditional urban traffic influence	severe soil contamination	high variability at the continental level	persistent historical pollution; exceeds safety norms

Note: N/A—not available/applicable.

**Table 2:** Influence of sampling period and extraction method on the physicochemical properties of the plant extracts (Two-way ANOVA).

Parameter	Factor	df	SS	F	*p*	Sig.
pH	Period	1.000	1.635	15.522	0.0002	***
pH	Method	3.000	2.557	8.090	9.29 × 10^−5^	***
Density	Period	1.000	0.0042	12.508	0.0007	***
Density	Method	3.000	0.0168	16.701	1.79 × 10^−8^	***
Refractive index	Period	1.000	31.282	44.137	3.72 × 10^−9^	***
Refractive index	Method	3.000	714.948	336.255	1.65 × 10^−44^	***
Conductivity	Period	1.000	105,337.500	1.819	0.1814	ns
Conductivity	Method	3.000	336,934.708	1.939	0.1301	ns

Note: ns = not significant, *** *p* < 0.0001.

**Table 3:** Comparative analysis of mineral and heavy metals content (mg/L) of *T. officinale* L. extracts.

*T. officinale* L. Sample	Radix		Herba		Flores		Mix	
Month/ Element	Oct	May	Oct	May	Oct	May	Oct	May
Iron (Fe 248)	3.896	4.473	1.769	3.805	6.172	7.168	8.113	8.160
Zinc (Zn 213)	2.503	0.3207	0.5675	0.2450	0.2926	0.4779	0.4285	0.3241
Manganese (Mn 279)	0.1397	0.3382	0.0512	0.3007	0.2462	0.3444	0.5648	0.4669
Copper (Cu324)	0.0505	0.0542	0.0154	0.0491	0.0784	0.1108	0.0798	1.087
Cadmium (Cd 228)	<LOD	<LOD	<LOD	<LOD	<LOD	<LOD	<LOD	<LOD
Lead (Pb 283)	<LOD	<LOD	<LOD	<LOD	<LOD	<LOD	<LOD	<LOD
Nickel (Ni 232)	<LOD	<LOD	<LOD	<LOD	<LOD	0.0030	0.0073	<LOD

**Table 4:** Bioactive principles identified in the extractive solution (adapted after [20]).

Bioactive Principle	Fraction A	Fraction B	Fraction C
**Radix**			
Gallic/catechin tannin	-	+	+
Steroli/triterpene	+	-	-
Polyuronides	-	-	+
Reducing compounds	-	+	+
Polyphenolcarboxylic acids	-	+	-
**Herba**			
Carotenoids	+	-	-
Fatty acids	+	-	-
Gallic/catechin tannin	-	+	+
Steroli/triterpene	+	-	-
Polyuronides	-	-	+
Reducing compounds	-	+	+
Polyphenolcarboxylic acids	-	+	-
**Flores**			
Carotenoids	+	-	-
Fatty acids	+	-	-
Gallic/catechin tannin	-	+	+
Steroli/triterpene	+	-	-
Flavonoside	-	+	-
Polyuronides	-	-	+
Reducing compounds	-	+	+
Polyphenolcarboxylic acids	-	+	-

**Table 5:** Analytical analysis from the HPLC screening of *Taraxacum officinale* Radix UAE selected extract.

Compound Identified	Identification Status	Monitored λ (nm)	Observed Rt (min)	Integrated Peak Area	Content (mg/100 g)
Gallic acid	Confirmed	274	0.920	879.42975	293.716
Kaempferol	Confirmed (Semi-quantitative)	280	1.062	1529.92175	111.136
Caftaric acid	Possible Match	310	2.158	139.41510	12.508
Chlorogenic acid	Confirmed	310	3.536	862.88184	33.790
Caffeic acid	Confirmed	310	4.607	63.68332	1.768
Syringic acid	Possible (Minor signal)	274	5.609	45.01352	1.933
Ellagic acid	Tentative (Suboptimal)	310	15.271	150.12852	9.756
Unassigned Derivative 1	Hydroxycinnamic/ Flavonoid	310/274/280	10.036	—	—
Unassigned Derivative 2	Hydroxycinnamic/ Flavonoid	310/274/280	11.740	—	—
Unassigned Derivative 3	Hydroxycinnamic/ Flavonoid	310/274/ 280	15.451– 5.625	—	—

**Table 6:** Analytical results from the HPLC screening of *Taraxacum officinale* Herba UAE selected extract.

Compound Identified	Verification Status	Acquisition λ (nm)	Observed Rt (min)	Integrated Peak Area	Calculated Content (mg/100 g)
Gallic acid	Confirmed	274	0.912	861.79492	287.816
Kaempferol	Confirmed (Semi-quantitative)	280	1.087	2287.41211	166.138
Luteolin/Luteol	Confirmed (Semi-quantitative)	280	1.176	724.71960	30.875
Syringic acid	Confirmed	274	4.918	481.48032	19.022
p-Coumaric acid	Confirmed	310	7.164	258.59274	6.379
Cinnamic acid	Confirmed	274	15.739	658.60907	72.897
Caffeic acid/Caffeic derivative	Possible Match	310	4.805	378.49637	9.905

**Table 7:** Analytical results from the HPLC screening of *Taraxacum officinale* Flores UAE selected extract.

Compound Identified	Identification Status	Monitored λ (nm)	Observed Rt (min.)	Integrated Peak Area	Content (mg/100 g)
Gallic acid	Confirmed	274	0.930	9014.74609	3015.559
Kaempferol	Confirmed (Semi-quantitative)	280	1.067	6586.32520	478.287
Luteol/Luteolin derivative	Possible (Semi-quantitative)	280	1.508	794.65869	33.799
Caftaric acid	Possible	310	2.412	371.08142	31.969
3-O-Methylgallic acid	Confirmed	274	2.460	672.12238	118.122
Chlorogenic acid/Caffeoylquinic isomer	Possible	310	3.189	580.02759	22.554
Syringic acid	Confirmed	274	4.983	1338.42578	52.573
p-Coumaric acid	Possible	310	7.035	268.89154	6.632

**Table 8:** Analytical results from the HPLC screening of *Taraxacum officinale* Mix UAE selected extract.

Compound Identified	Identification Status	Monitored λ (nm)	Observed Rt (min.)	Integrated Peak Area	Content (mg/100 g)
Gallic acid	Confirmed	274	0.916	1561.2336	521.828
Kaempferol	Confirmed (Semi-quantitative)	280	1.065	3739.9687	271.610
Luteol/Luteolin derivative	Possible (Semi-quantitative)	280	1.499	417.15884	18.019
Caftaric acid	Possible/Probable	310	2.147	1470.7454	124.347
3-O-Methylgallic acid	Confirmed	274	2.485	1045.8903	183.681
Chlorogenic acid	Confirmed	310	3.553	1444.7567	56.904
Caffeic acid	Confirmed	310	4.625	1287.3773	33.398
Syringic acid	Possible	274	5.218	264.97931	10.545
p-Coumaric acid	Confirmed	310	7.161	620.67834	15.298
Ellagic acid	Tentative (Suboptimal λ)	310	15.281	637.03540	58.113
Cinnamic acid	Tentative (Suboptimal λ)	310	15.905	410.78583	45.444
Major hydroxycinnamic derivative	Unquantified	310	16.262	1062.1695	—

**Table 9:** Quantitative and qualitative evaluation of antimicrobial activity expressed by the diameter of the inhibition zone (mm), minimum inhibitory concentration (MIC), minimum microbicidal concentration (MMC), and minimum inhibitory concentration of microbial adherence (MICMA)—October Harvest.

Sample	Analytical Parameter	*S. aureus* ATCC 25923	*E. faecalis* ATCC 29212	*P. aeruginosa* ATCC 27853	*E. coli* ATCC 25922	*C. albicans* ATCC 10231
Harvest in October						
Etanol 50%	IZD (mm)	0.00 ± 0.00	0.00 ± 0.00	0.00 ± 0.00	0.00 ± 0.00	0.00 ± 0.00
	MIC (µL/mL)	500	500	125	-	-
	MMC (µL/mL)	>500	>500	250	-	-
	MICMA (µL/mL)	>500	>500	125	-	-
Herba 50%	IZD (mm)	10.00 ± 1.00	11.66 ± 0.57	7.00 ± 1.00	0.00 ± 0.00	0.00 ± 0.00
	MIC (µL/mL)	250	250	250	-	-
	MMC (µL/mL)	>500	250	250	-	-
	MICMA (µL/mL)	250	250	250	-	-
Mix 50%	IZD (mm)	8.66 ± 0.57	10.33 ± 0.57	0.00 ± 0.00	0.00 ± 0.00	0.00 ± 0.00
	MIC (µL/mL)	250	250	-	-	-
	MMC (µL/mL)	500	250	-	-	-
	MICMA (µL/mL)	250	500	-	-	-
Flores 50%	IZD (mm)	7.64 ± 1.00	7.14 ± 0.57	0.00 ± 0.00	0.00 ± 0.00	0.00 ± 0.00
	MIC (µL/mL)	250	250	-	-	-
	MMC (µL/mL)	500	500	-	-	-
	MICMA (µL/mL)	250	250	-	-	-
Etanol 70%	IZD (mm)	0.00 ± 0.00	0.00 ± 0.00	0.00 ± 0.00	0.00 ± 0.00	0.00 ± 0.00
	MIC (µL/mL)	125	250	125	250	-
	MMC (µL/mL)	250	500	125	250	-
	MICMA (µL/mL)	125	250	62.5	125	-
Herba 70%	IZD (mm)	15.00 ± 1.00	13.00 ± 1.00	0.00 ± 0.00	8.00 ± 1.00	0.00 ± 0.00
	MIC (µL/mL)	250	250	-	250	-
	MMC (µL/mL)	500	>500	-	>500	-
	MICMA (µL/mL)	125	125	-	500	-
Mix 70%	IZD (mm)	14.66 ± 0.57	12.00 ± 1.00	10.00 ± 1.00	0.00 ± 0.00	0.00 ± 0.00
	MIC (µL/mL)	125	125	31.25	-	-
	MMC (µL/mL)	250	250	125	-	-
	MICMA (µL/mL)	125	125	62.5	-	-
Flores 70%	IZD (mm)	5.66 ± 0.57	11.00 ± 1.00	0.00 ± 0.00	0.00 ± 0.00	0.00 ± 0.00
	MIC (µL/mL)	62.5	62.5	-	-	-
	MMC (µL/mL)	125	125	-	-	-
	MICMA (µL/mL)	125	125	-	-	-
Herba Sox	IZD (mm)	11.33 ± 0.57	11.33 ± 0.57	10.00 ± 1.00	0.00 ± 0.00	0.00 ± 0.00
	MIC (µL/mL)	250	250	31.25	-	-
	MMC (µL/mL)	250	>500	125	-	-
	MICMA (µL/mL)	>500	125	62.5	-	-
Mix Sox	IZD (mm)	11.00 ± 1.00	12.00 ± 1.00	8.33 ± 1.15	0.00 ± 0.00	0.00 ± 0.00
	MIC (µL/mL)	125	250	62.5	-	-
	MMC (µL/mL)	500	250	250	-	-
	MICMA (µL/mL)	125	62.5	62.5	-	-
Flores Sox	IZD (mm)	11.00 ± 1.00	13.00 ± 1.00	0.00 ± 0.00	0.00 ± 0.00	0.00 ± 0.00
	MIC (µL/mL)	125	250	-	-	-
	MMC (µL/mL)	125	250	-	-	-
	MICMA (µL/mL)	125	62.5	-	-	-
Herba UAE	IZD (mm)	15.00 ± 1.00	15.33 ± 0.57	0.00 ± 0.00	0.00 ± 0.00	0.00 ± 0.00
	MIC (µL/mL)	125	125	-	-	-
	MMC (µL/mL)	>500	>500	-	-	-
	MICMA (µL/mL)	62.5	62.5	-	-	-
Mix UAE	IZD (mm)	15.00 ± 1.00	16.00 ± 1.00	0.00 ± 0.00	0.00 ± 0.00	0.00 ± 0.00
	MIC (µL/mL)	250	125	-	-	-
	MMC (µL/mL)	250	>500	-	-	-
	MICMA (µL/mL)	125	62.5	-	-	-
Flores UAE	IZD (mm)	0.00 ± 0.00	10.33 ± 0.57	12.00 ± 0.57	12.00 ± 1.00	0.00 ± 0.00
	MIC (µL/mL)	-	125	62.5	125	-
	MMC (µL/mL)	-	125	125	250	-
	MICMA (µL/mL)	-	62.5	62.5	31.25	-
Gentamicin	IZD (280 µg/mL, mm)	18.00 ± 1.41	0.00 ± 0.00	12.50 ± 0.71	19.00 ± 0.71	-
	MIC (µL/mL)	1.09	140	4.38	17.5	-
	MMC (µL/mL)	17.5	4.38	8.75	70	-
	MICMA (µL/mL)	17.5	70	4.38	17.5	-
Ketoconazole	IZD (140 µg/mL, mm)	-	-	-	-	6.0 ± 1.41
	MIC (µL/mL)	-	-	-	-	8.75
	MMC (µL/mL)	-	-	-	-	70
	MICMA (µL/mL)	-	-	-	-	17.5

Note: The blue highlighting indicates the variants for which the MICMA value was lower than the corresponding MIC value. The orange highlighting indicates MIC values lower than those of the corresponding solvent control, while the green highlighting indicates MMC values lower than those of the corresponding solvent control.

**Table 10:** Quantitative and qualitative evaluation of antimicrobial activity expressed by the diameter of the inhibition zone (mm), minimum inhibitory concentration (MIC), minimum microbicidal concentration (MMC), and minimum inhibitory concentration of microbial adherence (MICMA)—May harvest.

Sample	Analytical Parameter	*S. aureus* ATCC 25923	*E. faecalis* ATCC 29212	*P. aeruginosa* ATCC 27853	*E. coli* ATCC 25922	*C. albicans* ATCC 10231
Harvest in May						
Etanol 50%	IZD (mm)	0.00 ± 0.00	0.00 ± 0.00	0.00 ± 0.00	0.00 ± 0.00	0.00 ± 0.00
	MIC (µL/mL)	500	500	125	125	125
	MMC (µL/mL)	>500	>500	250	250	500
	MICMA (µL/mL)	>500	>500	125	250	250
Radix 50%	IZD (mm)	7.66 ± 1.15	7.33 ± 0.57	3.33. ± 0.57	0.00 ± 0.00	0.00 ± 0.00
	MIC (µL/mL)	125	500	62.5	-	-
	MMC (µL/mL)	500	>500	125	-	-
	MICMA (µL/mL)	250	500	125	-	-
Herba 50%	IZD (mm)	0.00 ± 0.00	8.33 ± 0.57	6.00 ± 0.57	0.00 ± 0.00	0.00 ± 0.00
	MIC (µL/mL)	-	250	125	-	-
	MMC (µL/mL)	-	250	250	-	-
	MICMA (µL/mL)	-	250	250	-	-
Mix 50%	IZD (mm)	5.66 ± 0.57	9.00 ± 1.00	4.00 ± 1.00	0.00 ± 0.00	0.00 ± 0.00
	MIC (µL/mL)	125	250	125	-	-
	MMC (µL/mL)	500	500	250	-	-
	MICMA (µL/mL)	250	250	125	-	-
Etanol 70%	IZD (mm)	0.00 ± 0.00	0.00 ± 0.00	0.00 ± 0.00	0.00 ± 0.00	0.00 ± 0.00
	MIC (µL/mL)	125	250	125	250	125
	MMC (µL/mL)	250	500	125	250	500
	MICMA (µL/mL)	125	250	62.5	125	125
Herba 70%	IZD (mm)	7.00 ± 1.00	6.00 ± 1.00	0.00 ± 0.00	5.00 ± 1.00	0.00 ± 0.00
	MIC (µL/mL)	250	250	-	250	-
	MMC (µL/mL)	500	>500	-	>500	-
	MICMA (µL/mL)	125	125	-	500	-
Mix 70%	IZD (mm)	8.66 ± 1.15	8.00 ± 1.00	0.00 ± 0.00	0.00 ± 0.00	3.00 ± 1.00
	MIC (µL/mL)	125	250	-	-	125
	MMC (µL/mL)	250	500	-	-	250
	MICMA (µL/mL)	125	125	-	-	125
Flores 70%	IZD (mm)	4.33 ± 0.57	6.00 ± 1.00	0.00 ± 0.00	0.00 ± 0.00	0.00 ± 0.00
	MIC (µL/mL)	62.5	62.5	-	-	-
	MMC (µL/mL)	250	125	-	-	-
	MICMA (µL/mL)	125	125	-	-	-
Radix Sox	IZD (mm)	8.33 ± 0.57	3.00 ± 1.00	2.00 ± 1.00	2.00 ± 1.00	0.00 ± 0.00
	MIC (µL/mL)	250	250	62.5	125	-
	MMC (µL/mL)	250	>500	250	250	-
	MICMA (µL/mL)	>500	250	62.5	250	-
Herba Sox	IZD (mm)	9.33 ± 0.57	9.33 ± 0.57	9.00 ± 1.00	0.00 ± 0.00	0.00 ± 0.00
	MIC (µL/mL)	250	250	62.5	-	-
	MMC (µL/mL)	250	>500	250	-	-
	MICMA (µL/mL)	>500	125	62.5	-	-
Mix Sox	IZD (mm)	10.00 ± 1.00	7.00 ± 1.00	3.33 ± 0.57	0.00 ± 0.00	0.00 ± 0.00
	MIC (µL/mL)	62.5	250	62.5	-	-
	MMC (µL/mL)	250	500	250	-	-
	MICMA (µL/mL)	125	250	62.5	-	-
Flores Sox	IZD (mm)	7.33 ± 1.15	9.00 ± 1.00	4.00 ± 1.00	0.00 ± 0.00	0.00 ± 0.00
	MIC (µL/mL)	125	125	31.25	-	-
	MMC (µL/mL)	125	250	125	-	-
	MICMA (µL/mL)	125	62.5	62.5	-	-
Radix UAE	IZD (mm)	8.00 ± 1.00	5.33 ± 0.57	2.00 ± 1.00	0.00 ± 0.00	0.00 ± 0.00
	MIC (µL/mL)	125	125	62.5	-	-
	MMC (µL/mL)	250	500	500	-	-
	MICMA (µL/mL)	62.5	250	62.5	-	-
Herba UAE	IZD (mm)	9.00 ± 1.00	11.33 ± 1.15	5.33 ± 0.57	4.33 ± 0.57	0.00 ± 0.00
	MIC (µL/mL)	125	250	125	62.5	-
	MMC (µL/mL)	500	>500	500	250	-
	MICMA (µL/mL)	62.5	62.5	62.5	31.25	-
Flores UAE	IZD (mm)	5.00 ± 1.00	3.33 ± 0.57	3.00 ± 1.00	2.33 ± 0.57	4.33 ± 0.57
	MIC (µL/mL)	62.5	250	62.5	62.5	125
	MMC (µL/mL)	250	500	250	250	500
	MICMA (µL/mL)	125	125	125	62.5	125
Mix UAE	IZD (mm)	15.00 ± 1.00	8.00 ± 1.00	10.00 ± 1.00	0.00 ± 0.00	0.00 ± 0.00
	MIC (µL/mL)	125	125	62.5	-	-
	MMC (µL/mL)	250	500	500	-	-
	MICMA (µL/mL)	125	62.5	62.5	-	-

Note: The blue highlighting indicates the variants for which the MICMA value was lower than the corresponding MIC value. The orange highlighting indicates MIC values lower than those of the corresponding solvent control, while the green highlighting indicates MMC values lower than those of the corresponding solvent control.

**Table 11:** Non-parametric statistical analysis of extract antimicrobial efficacy.

Test	H/U	*p*	Sig.
Across 5 strains	231.84	5.29 × 10^−49^	***
Gram-positive vs. Gram-negative (Mann–Whitney)	21.242	6.56 × 10^−31^	***
Season October vs. May (Mann–Whitney)	5012	0.0000	***
Method (Kruskal–Wallis)	6.57	0.087	ns

Note: H/U = test statistic value: H (Kruskal-Wallis test statistic), U (Mann-Whitney U test statistic); *p*-value = probability value; Sig = Statistical Significance; ns = not significant; *** *p* < 0.001.

**Table 12:** Classification of induced effects, according to LC_50_, by quantifying toxicity.

*T. officinale* Extract	LC_50_ (mg/mL)	Effect
Radix	>1000 (325,316.36)	nontoxic
Herba	76.15	nontoxic
Flores	66.73	nontoxic
Mix	>500 (524.93)	nontoxic

Note: Clarkson’s toxicity criterion (extracts with LC_50_ above 1000 μg/mL are non-toxic; LC_50_ of 500–1000 μg/mL are low toxic; LC_50_ of 100–500 μg/mL are medium toxic; while extracts LC_50_ with LC_50_ of 0–100 μg/mL are highly toxic).

**Table 13:** Differences regarding RGL (µm) in case of larvae exposed to *T. officinale* L. extracts, after 4 days of exposure; statistical significance was calculated according to the Tukey B test (*p* < 0.005).

Groups	Difference	Test Statistic	*p*-Value	Significant
Control vs. Radix	−350.45	31.38	8.92 × 10^−5^	Yes
Control vs. Herba	−138.72	12.42	4.72 × 10^−5^	Yes
Control vs. Flores	−139.45	12.49	3.45 × 10^−5^	Yes
Control vs. Mix	−135.58	12.14	7.53 × 10^−5^	Yes
Herba vs. Radix	−211.73	18.96	4.72 × 10^−5^	Yes
Herba vs. Mix	−3.15	0.28	0.92	No
Flores vs. Radix	−211	18.89	7.53 × 10^−5^	Yes
Flores vs. Herba	−0.73	0.07	0.98	No
Flores vs. Mix	−3.88	0.35	0.98	No
Mix vs. Radix	−214.88	19.24	3.45 × 10^−5^	Yes

**Table 14:** Optimal conditions for extracting bioactive compounds from *Taraxacum officinale* L.

Target Compound	Optimal Season	Optimal Plant Part	Optimal Extraction Method
Tannins	Autumn	Roots	Ultrasound (UAE)
Flavonoids	Spring	Flowers	Ultrasound (UAE)
Ascorbic acid	Spring	Aerial Parts	50% Maceration/UAE
Carotenoid	Spring	Flowers	Soxhlet

**Table 15:** Binary gradient elution profile for multi-component phenolic separation.

No.	Time (min)	Solution A (%, *v*/*v*)	Solution B (%, *v*/*v*)
1	0–13	90	10
2	13	78	22
3	14	60	40
4	17	60	40
5	17.5	90	10
6	22	90	10

## Data Availability

Data is contained within the article or Supplementary Material.

## Supplementary Materials

The following supporting information can be downloaded at: https://www.mdpi.com/article/10.3390/molecules31142549/s1, Figure S1: AAS calibration curves for minerals (Cu, Mn, Zn, Fe) and heavy metals (Cd, Pb, Ni); Figure S2: RP-HPLC-DAD chromatograms (λ = 310 nm) of *Taraxacum officinale* Radix UAE extract; Figure S3: RP-HPLC-DAD chromatograms (λ = 230 nm) of *Taraxacum officinale* Herba UAE extract; Figure S4: RP-HPLC-DAD chromatograms (λ = 310 nm) of *Taraxacum officinale* Flores UAE extract; Figure S5: RP-HPLC-DAD chromatograms (λ = 310 nm) of *Taraxacum officinale* Mix UAE extract; Figure S6: The antimicrobial effect of 1—Herba 50%, 2—Mix 50%, 3—Flores 50%, 4—Flores 70%, 5—Mix 70%, 6—Herba Sox, 7—Mix Sox, 8—Flores UAE against *Staphylococcus aureus*, *Enterococcus faecalis*, *Pseudomonas aeruginosa*, *Escherichia coli* and *Candida albicans*; Figure S7: Exposed the larval stage of *Artemia salina* at 24 h (Stage I) and 72 h (Stage III).

## Abbreviations

The following abbreviations are used in this manuscript:

DW	dry weight
Et 50%	50:50 (*v*/*v*) ethanolic extract
Et 70%	70:30 (*v*/*v*) ethanolic extract
LPS	lipopolysaccharide
MIC	minimum inhibitory concentration
MBC	minimum microbicidal concentration
MICMA	minimum inhibitory concentration of microbial adherence
UAE	ultrasound-assisted extraction
