# Supplementary material for: Phytochemical Dynamics and Antimicrobial Efficacy of Dandelion (Taraxacum officinale L.) from Central Plateau of Moldova, Romania
Source: Molecules. 2026 Jul 22;31(14):2549. doi: 10.3390/molecules31142549 (PMC13416477; doi:10.3390/molecules31142549)
Supplement: Supplementary file 1 [file molecules-31-02549-s001.zip › molecules-4396766-supplementary_v6.pdf]

Supplementary Materials

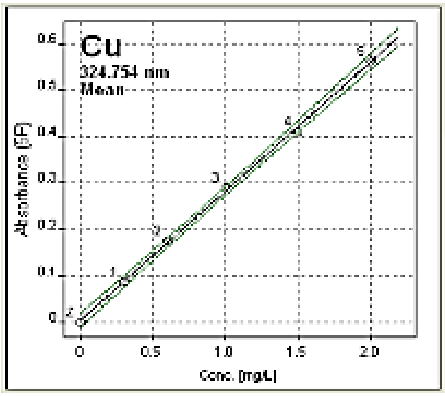

Calibration data

R²(adj.): 0.998146790
Slope: 0.2778000 Abs./mg/L
Method SD: 0.0281131 mg/L
Char.conc.: 0.0156947 mg/L/1%A
y=a+bx
a=0.0048363 b=0.2778000

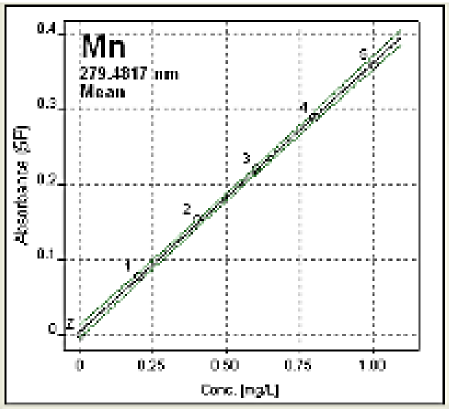

Calibration data

R²(adj.): 0.998659346
Slope: 0.3561638 Abs./mg/L
Method SD: 0.0118694 mg/L
Char.conc.: 0.0122416 mg/L/1%A
y=a+bx
a=0.0050100 b=0.3561638

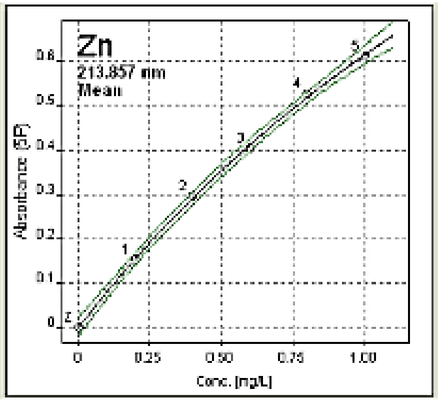

Calibration data

R²(adj.): 0.998533650
Slope: 0.8353544 Abs./mg/L
Method SD: 0.0119756 mg/L
Char.conc.: 0.0052193 mg/L/1%A
y=(a+bx)/(1+cx)
a=-.0003577 b=0.8352264 c=0.3577993

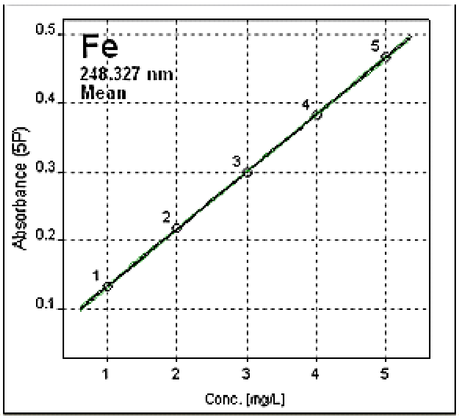

Calibration data

R²(adj.): 0.999905337
Slope: 0.0827824 Abs./mg/L
Method SD: 0.0125610 mg/L
Char.conc.: 0.0526682 mg/L/1%A
y=a+bx
a=0.0515379 b=0.0827824

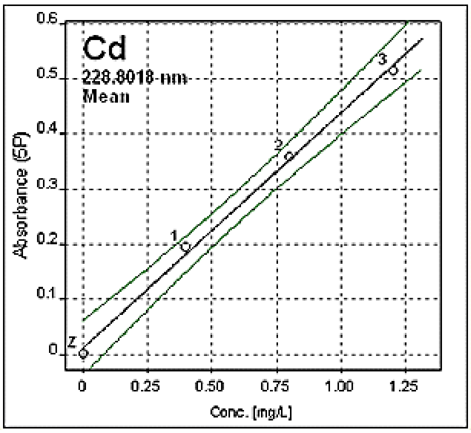

Calibration data

R²(adj.):	0.992692622	
Slope:	0.4275206 Abs./mg/L	
Method SD:	0.0312521 mg/L	
Char.conc.:	0.0101983 mg/L/1%A	
y=a+bx		
a=0.0111676	b=0.4275206	

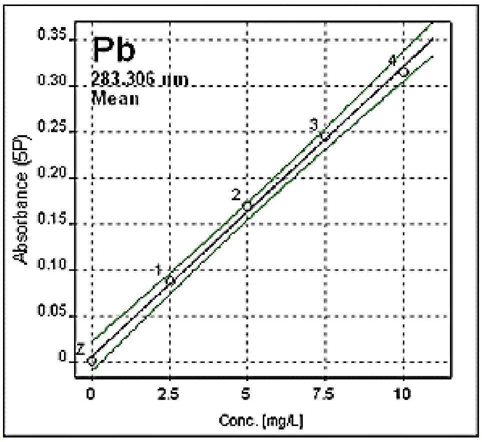

Calibration data

R²(adj.):	0.996138267	
Slope:	0.0314956 Abs./mg/L	
Method SD:	0.2007591 mg/L	
Char.conc.:	0.1384322 mg/L/1%A	
y=a+bx		
a=0.0063773	b=0.0314956	

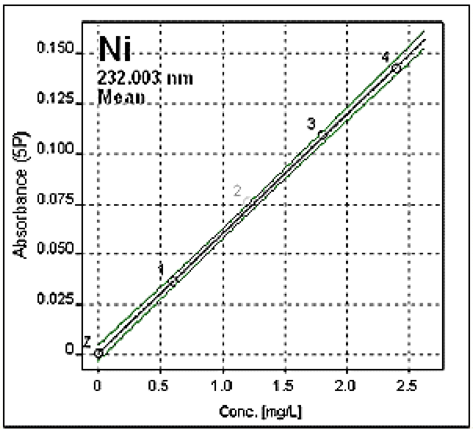

Calibration data

R²(adj.):	0.999431813	
Slope:	0.0593700 Abs./mg/L	
Method SD:	0.0184656 mg/L	
Char.conc.:	0.0734378 mg/L/1%A	
y=a+bx		
a=0.0009802	b=0.0593700	

Figure S1. AAS calibration curves for minerals (Cu, Mn, Zn, Fe) and heavy metals (Cd, Pb, Ni)

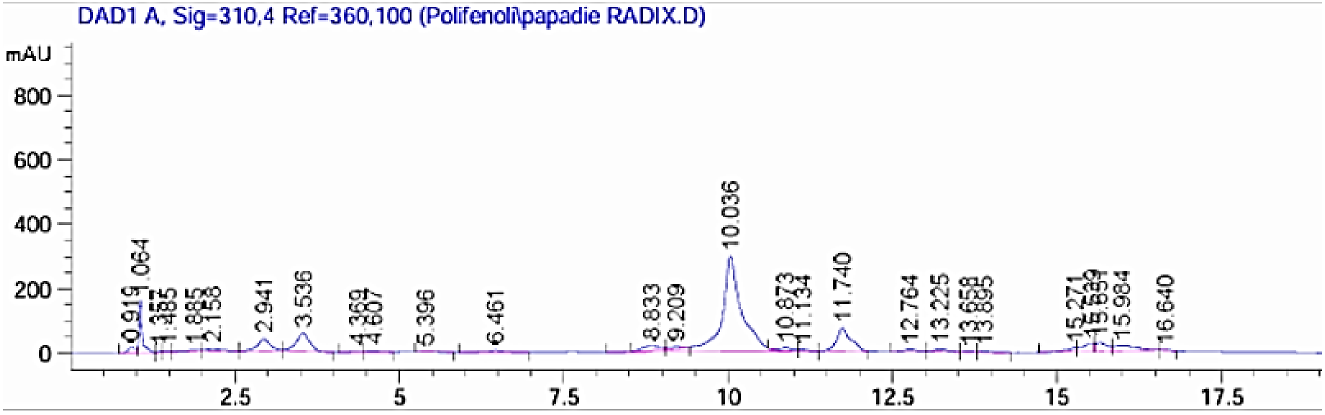

Figure S2. RP-HPLC-DAD chromatograms (**λ** = 310 nm) of *Taraxacum officinale* Radix UAE extract

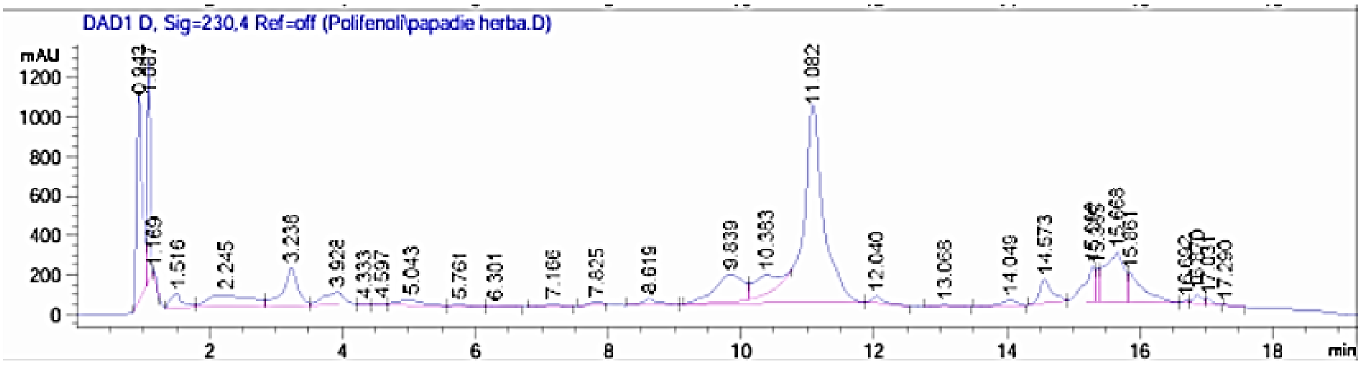

Figure S3. RP-HPLC-DAD chromatograms (**λ** = 230 nm) of *Taraxacum officinale* Herba UAE extract

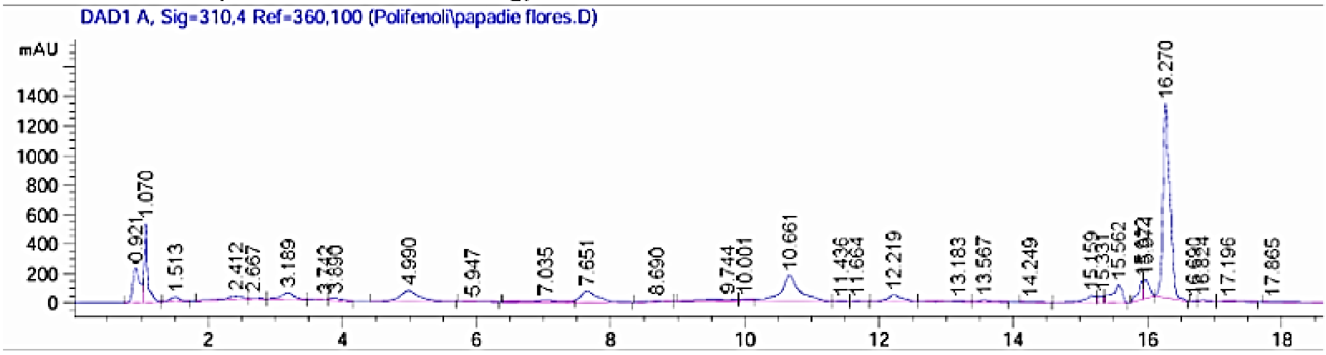

Figure S4. RP-HPLC-DAD chromatograms (**λ** = 310 nm) of *Taraxacum officinale* Flores UAE extract

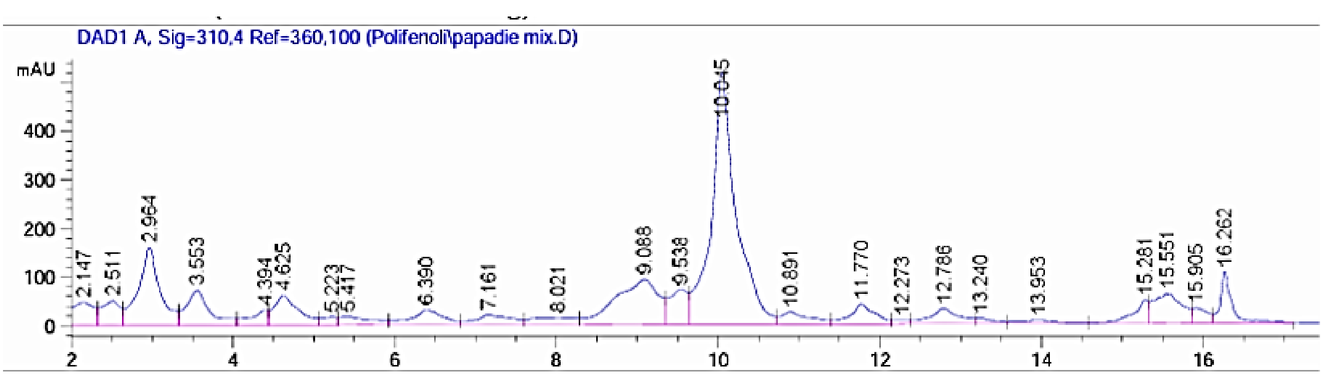

Figure S5. RP-HPLC-DAD chromatograms (λ = 310 nm) of *Taraxacum officinale* Mix UAE extract

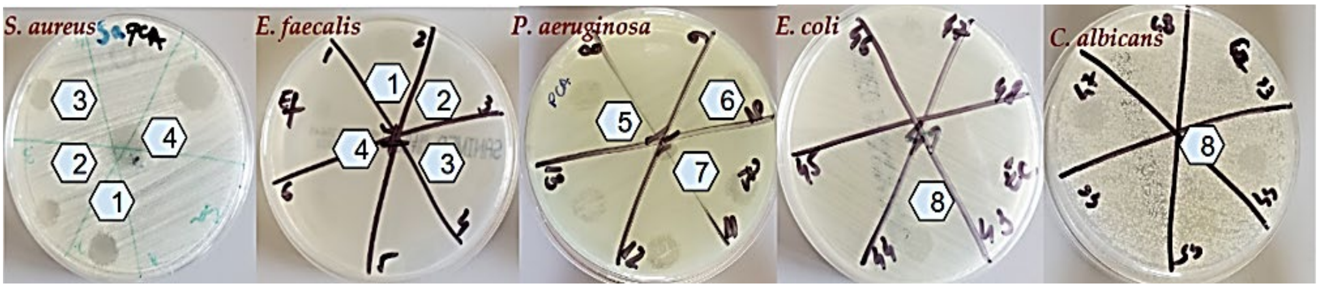

Figure S6. The antimicrobial effect of 1 - Herba 50%, 2 - Mix 50%, 3 – Flores 50%, 4 - Flores 70%, 5 – Mix 70%, 6 – Herba Sox, 7 – Mix Sox, 8 - Flores UAE against *Staphylococcus aureus*, *Enterococcus faecalis*, *Pseudomonas aeruginosa*, *Escherichia coli*, and *Candida albicans*

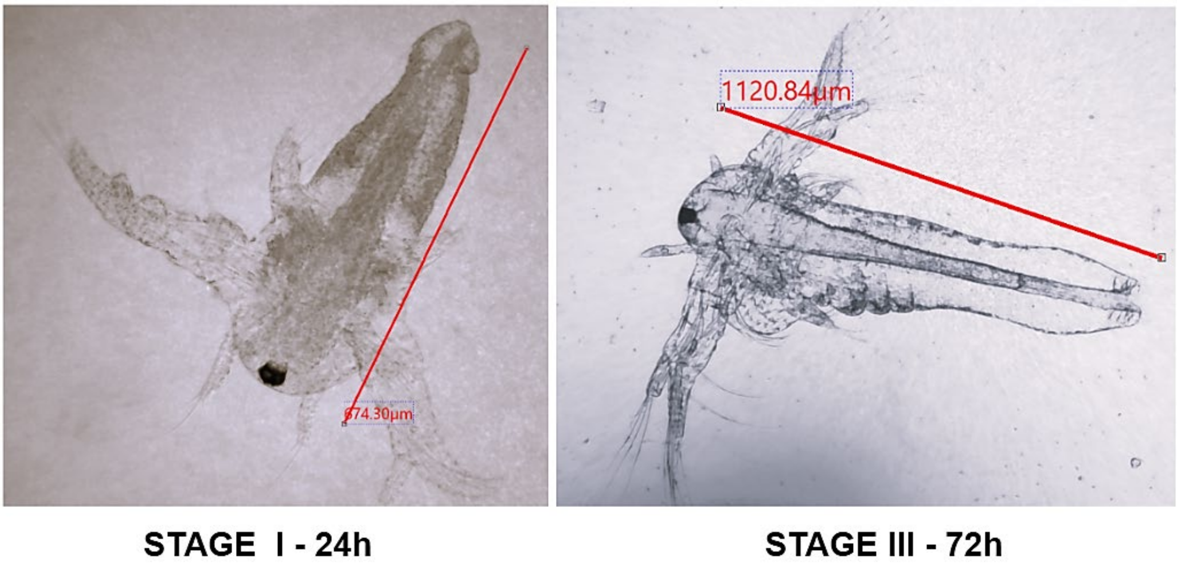

Figure S7. Exposed the larval stage of *Artemia salina* at 24 hours (Stage I) and 72 hours (Stage III)
